# Reconstitution of recombinant human CCR4-NOT reveals molecular insights into regulated deadenylation

**DOI:** 10.1038/s41467-019-11094-z

**Published:** 2019-07-18

**Authors:** Tobias Raisch, Chung-Te Chang, Yevgen Levdansky, Sowndarya Muthukumar, Stefan Raunser, Eugene Valkov

**Affiliations:** 1 Department of Biochemistry, Max Planck Institute for Developmental Biology, Max-Planck-Ring 5, 72076 Tübingen, Germany; 2 Department of Structural Biochemistry, Max Planck Institute of Molecular Physiology, Otto-Hahn-Strasse 11, 44227 Dortmund, Germany

**Keywords:** Gene expression, Multienzyme complexes, RNA, RNA decay

## Abstract

CCR4-NOT is a conserved multiprotein complex which regulates eukaryotic gene expression principally via shortening of poly(A) tails of messenger RNA or deadenylation. Here, we reconstitute a complete, recombinant human CCR4-NOT complex. Our reconstitution strategy permits strict compositional control to test mechanistic hypotheses with purified component variants. CCR4-NOT is more active and selective for poly(A) than the isolated exonucleases, CCR4a and CAF1, which have distinct deadenylation profiles in vitro. The exonucleases require at least two out of three conserved non-enzymatic modules (CAF40, NOT10:NOT11 or NOT) for full activity in CCR4-NOT. CAF40 and the NOT10:NOT11 module both bind RNA directly and stimulate deadenylation in a partially redundant manner. Linear motifs from different RNA-binding factors that recruit CCR4-NOT to specific mRNAs via protein-protein interactions with CAF40 can inhibit bulk deadenylation. We reveal an additional layer of regulatory complexity to the human deadenylation machinery, which may prime it either for general or target-specific degradation.

## Abstract (web summary)

The CCR4-NOT complex shortens poly(A) tails of messenger RNAs. By biochemical reconstitution of the entire human CCR4-NOT complex, the authors show the stimulatory roles of non-enzymatic subunits and the importance of the interaction between CAF40 and RNA binding proteins in targeted deadenylation.

## Introduction

The poly(A) tails at 3′ ends of eukaryotic mRNAs are crucial for their cytoplasmic stability and to enhance the initiation of translation. Newly synthesized metazoan mRNAs possess long poly(A) tails^1^, and following export to the cytoplasm the tails are reported to be ~60–80 nucleotides on average at steady state^2^. Poly(A) tails are also important for translational efficiency at the embryonic stage^2^ and the length of the poly(A) tail was reported to be correlated with translational efficiency^3^. The multisubunit CCR4-NOT complex is principally responsible for efficient processive shortening of poly(A) tails, or deadenylation, in addition to other functions^4–7^. In addition to its role in bulk mRNA decay, CCR4-NOT can also catalyze the deadenylation or promote translational repression of specific mRNA targets to which it is recruited by RNA binding proteins, such as Nanos, Roquin and Puf/Pumilio proteins^8–13^. In animals, CCR4-NOT functions in cytoplasmic microRNA-mediated gene silencing via a direct interaction with the GW182/TNRC6 proteins^8,14,15^, as well as in the nuclear miRNA-mediated gene silencing, which is critical for stem cell differentiation^16^.

**Fig. 1:**
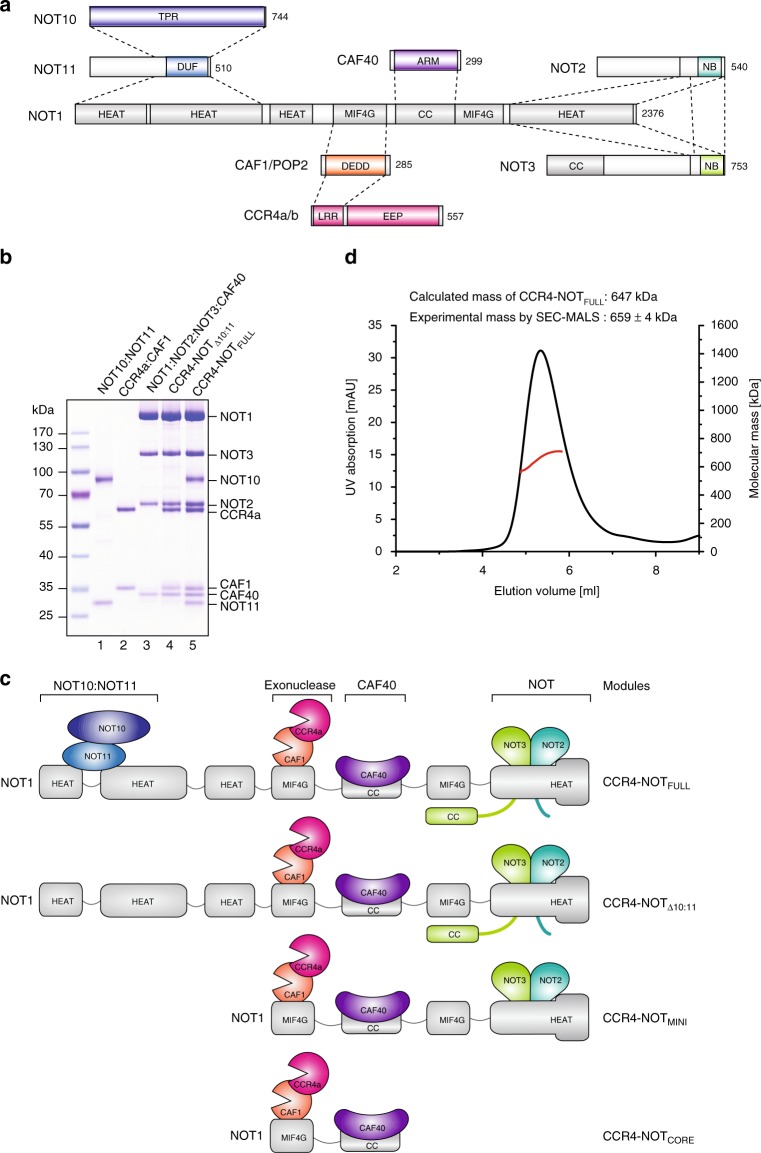
Architecture and reconstitution of the human CCR4-NOT complex. **a** Schematic representation of the human CCR4-NOT complex. Domains are indicated and known interactions between domains are shown by dashed lines. HEAT: domain consisting of α-helical HEAT-like repeats; MIF4G: HEAT-like domain with structural similarity to the middle domain of eIF4G; DUF: domain of unknown function 2363; TPR: predicted domain consisting of α-helical TPR-like repeats; ARM: domain consisting of α-helical armadillo repeats; EEP: endonuclease/exonuclease/phosphatase family member; LRR: Leucine-rich repeats; DEDD: exonuclease of the DEDD family; CC: predicted coiled-coil domain; NB NOT-box domain. **b** Coomassie-stained gel with the purified recombinant NOT10:NOT11, CCR4a:CAF1 and NOT1:NOT2:NOT3:CAF40 complexes used for modular reconstitution, and the resulting assembled CCR4-NOT_Δ10:11_ and CCR4-NOT_FULL_ complexes. Source data are provided as a Source Data file. **c** Schematic representation of the reconstituted complexes used in this study. CCR4-NOT_Δ10:11_ lacks the NOT10:NOT11 heterodimer. In CCR4-NOT_MINI_ the N-terminal parts of NOT1, NOT2, and NOT3 are truncated. CCR4-NOT_CORE_ comprises just two structured domains of NOT1 with the docked exonucleases CAF1 and CCR4, as well as CAF40. **d** Size exclusion chromatography (SEC) elution profile of the reconstituted CCR4-NOT_FULL_ complex with multi-angle laser light scattering (MALS) profile shown in red for the protein peak. Theoretical and experimentally derived mass is indicated

The CCR4-NOT complex consists of two exonucleases, CCR4 and CAF1, as well as the non-enzymatic proteins NOT1, NOT2, NOT3, and CAF40 which are conserved in all eukaryotes (Fig. 1a). NOT1 is an essential subunit of CCR4-NOT and functions as a scaffold on which other subunits and modules dock^17–19^. The highly conserved CAF40 and NOT2/3 subunits act as protein–protein interaction platforms for sequence-specific RNA-binding proteins^10,11,13,20^. The exonucleases CCR4a (NOT6) and CCR4b (NOT6L) are vertebrate orthologs of the yeast Ccr4 whilst NOT7 and NOT8, which belong to a large family called Caf1, are vertebrate orthologs of the yeast Pop2, respectively^21–23^. Ccr4 is the key functional deadenylase in yeast^24^, whereas CAF1 was shown to be crucial for deadenylation in nematodes^25^ and in *Drosophila* S2 cells^26^. In human cells, CAF1 is important for microRNA-mediated or small interfering RNA-mediated deadenylation^27^. Recently it was shown that CCR4 is the dominant deadenylase of the human and yeast CCR4-NOT complexes on tails coated with poly(A) binding protein PABPC1 whereas CAF1 is blocked^7,28^. Other species-specific compositional differences relate to the NOT4 subunit, which functions as an E2-dependent RING E3 ligase. It is stably incorporated within the yeast Ccr4-Not but not in *Drosophila* S2 and human cells^18,21–23,29,30^. The largely uncharacterized subunits NOT10 and NOT11 are widely conserved in eukaryotes, except for yeast, and interact stably with the NOT1 subunit (Fig. 1a)^19,23,31^.

One long-standing question regarding the CCR4-NOT complex has been that of the role of the non-enzymatic subunits of CCR4-NOT in supporting deadenylation and/or substrate selectivity. Previous results showed that those subunits support target-specific deadenylation by providing binding sites for factors which recruit CCR4-NOT to specific transcripts^10,11,13,20,32–34^, but the question whether other, more direct mechanisms of regulating the nucleases might exist, remained unanswered. Recombinant fission yeast Ccr4-Not complex is strikingly more active and sequence-selective than the Ccr4/Caf1 exonucleases alone^35^. However, the species-specific differences in the subunit composition mean that these results are not directly transferable to the mammalian complex. Hence, in order to investigate the molecular mechanisms of mammalian CCR4-NOT, access to reproducible, active and compositionally defined preparation is essential. However, despite intense efforts, the isolation of fully assembled and compositionally homogeneous mammalian CCR4-NOT complex has not yet been reported. This limits our mechanistic understanding to studies with isolated subunits and subcomplexes^7,32,33,36–41^.

Here, we describe a procedure to reconstitute the human CCR4-NOT complex using a stepwise assembly of purified recombinant components. Our modular assembly approach permits a simple exchange of variants such as truncated constructs and mutants to rapidly exert full compositional control to test mechanistic hypotheses. We observe that the intact human CCR4-NOT has substantially increased deadenylation activity and sequence selectivity in vitro compared to the exonucleases alone. However, this is not strongly influenced by the sequence composition of the segment preceding the poly(A) tail. Several non-enzymatic modules directly stimulate deadenylation by supporting RNA binding in an apparently redundant manner. Binding of RNA-binding proteins to the CAF40 subunit, in turn, inhibits this intrinsic stimulation of deadenylation. Biochemical reconstitution of human CCR4-NOT presents a strikingly holistic view of this complex in which multiple subunits act in concert to regulate deadenylation.

## Results

### Production of assembly intermediates of human CCR4-NOT

Functional native CCR4-NOT has been isolated from yeast, *Drosophila* and human cells^18,22,23,35^. However, isolation of native complexes is challenging, time-consuming and results in compositionally heterogeneous preparations, which are not manipulable or easily tractable for biochemical study. To solve this problem, we focused our approach on in vitro reconstitution with highly purified, recombinant human proteins. First, we generated a single recombinant baculovirus with genes encoding all eight subunits (CCR4a, CAF1, NOT1, NOT2, NOT3, CAF40, NOT10, and NOT11; Fig. 1a; Supplementary Table 1) using the MultiBac system^42,43^. However, production of the entire complex in insect cells was hampered by low yields and poor subunit stoichiometry. We then revised our approach to co-produce subcomplexes with subsequent reconstitution. We generated a baculovirus comprising the full-length NOT1 together with a minimal set of three full-length proteins (NOT2, NOT3, and CAF40; Supplementary Fig. 1a) rationalized by observations that these four subunits are mutually stabilizing^33,34,39,44^. A decahistidine tag fused to the N-terminus of the NOT1 scaffold subunit was used for metal affinity purification with typical yields of 4–5 mg per liter of insect cell culture (Supplementary Fig. 1b). Remarkably, we observed almost no proteolytic degradation of NOT1, NOT2, and NOT3 despite their size and presence of low-complexity regions (Fig. 1b, lane 3).

The heterodimers of NOT10:NOT11 and the CCR4a:CAF1 exonucleases were recombinantly produced in bacteria (Supplementary Fig. 1c–f). A heterodimer of CCR4a and CAF1 full-length exonucleases was purified by metal affinity capture followed by size exclusion chromatography and a final high-resolution anion exchange step to obtain a complex of 1:1 stoichiometry (Supplementary Fig. 1c, d). Previous work indicated that the C-terminal portion of NOT11 (residues 257-498) was sufficient to form a stable trimeric complex with an N-terminal region of NOT1, as well as NOT10^19,20^. This NOT11 construct including a hexahistidine tag was co-produced in bacteria with NOT10 (residues 25-707) fused to an N-terminal maltose-binding protein (MBP) tag for stability (Supplementary Fig. 1e). Metal affinity capture was followed by proteolytic removal of MBP and subsequent size exclusion chromatography (Supplementary Fig. 1f). The purity of the heterodimeric subcomplexes was confirmed by SDS-PAGE (Fig. 1b, lanes 1 and 2).

### Modular reconstitution of the CCR4-NOT complex

We observed that an intact CCR4-NOT comprising eight subunits, which we termed CCR4-NOT_FULL_ (Fig. 1a, c), could be simply and efficiently assembled from three purified subcomplexes by incubating the NOT1:NOT2:NOT3:CAF40 subcomplex produced in insect cells with a two-fold molar excess of CCR4a:CAF1 and NOT10:NOT11 heterodimers on ice for two hours followed by a separation of the mixture of complexes by size exclusion chromatography (Supplementary Fig. 2a, b). SDS-PAGE analysis with Coomassie staining confirmed that the reconstituted CCR4-NOT_FULL_ comprised all eight protein subunits in an equimolar stoichiometric ratio, as expected (Fig. 1b, lane 5). This was further corroborated by multiangle light scattering coupled with a size exclusion column (SEC-MALS), which indicated that the estimated molecular mass of the peak corresponding to the eight-subunit complex (659 +/− 4 kDa [+/− value indicates technical measurement uncertainty]) was in close agreement with the calculated mass of 647 kDa and the complex was monodisperse in solution (Fig. 1d). Thus, we established a simple and reproducible modular strategy to reconstitute biochemically tractable preparations of human CCR4-NOT with a strictly defined subunit composition.

### CCR4-NOT_FULL_ is more active and specific than CCR4a:CAF1

**Fig. 2:**
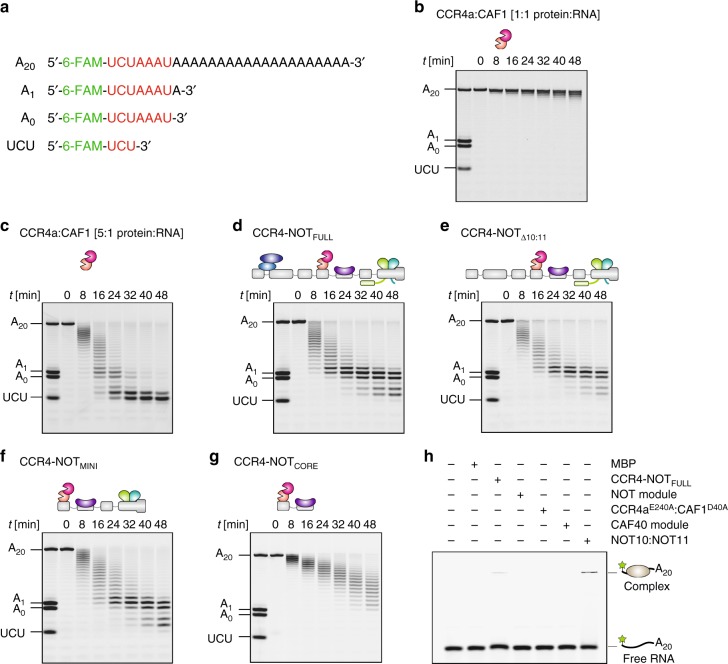
The contribution of the non-enzymatic modules towards activity. **a** The sequence of the synthetic 7-mer-A_20_ RNA substrate, which consists of a 5′ end 6-carboxyfluorescein (6-FAM, green) fluorophore, a 7-mer RNA body (red) and a tail of 20 adenosines (A_20_, black). Positional marker RNAs are also depicted. **b** Deadenylation assay time course experiment with the 7-mer-A_20_ RNA substrate and the CCR4a:CAF1 exonuclease heterodimer in equimolar ratio (50 nM) demonstrating low catalytic activity. **c** Time course assay as in **b**, but with a 5-fold molar excess of CCR4a:CAF1 (250 nM) over 7-mer-A_20_ RNA (50 nM). Under these conditions, the deadenylation reaction is rapid. **d**–**f** Deadenylation assays with 50 nM 7-mer-A_20_ RNA and equimolar concentrations of the CCR4-NOT_FULL_ (**d**), CCR4-NOT_Δ10:11_ (**e**), and CCR4-NOT_MINI_ (**f**) complexes. All three complexes produce very similar deadenylation patterns and display enhanced activity compared to the CCR4a:CAF1 heterodimer under the same conditions (**b**). **g** Deadenylation assay with 7-mer-A_20_ RNA and the CCR4-NOT_CORE_ complex in equimolar ratio (50 nM). The deadenylation reaction is slower than with the larger reconstituted complexes (**d**–**f**) but visibly enhanced compared to the CCR4a:CAF1 heterodimer (**b**). **h** Electrophoretic mobility shift assay (EMSA) with 100 nM the 7-mer-A_20_ RNA and 1 µM CCR4-NOT_FULL_ (CCR4a^E240A^:CAF1^D40A^) and subcomplexes. The CCR4-NOT_FULL_ (CCR4a^E240A^:CAF1^D40A^) complex and the NOT10:NOT11 heterodimer both bind the RNA as visible from the shift of the RNA band to apparent higher molecular weight in the respective lanes. The upshifted protein-RNA complexes did not enter the gel. Source data for panels **b**–**h** are provided as a Source Data file. All assays shown in this figure and other figures are supported by technical, as well as biological replicates

To assess the deadenylation activity of the reconstituted human CCR4-NOT complex we utilized a synthetic RNA substrate of seven nucleotides (5′-UCUAAAU-3′) followed by a polyadenosine tail of 20 nucleotides (A_20_) and labeled with fluorescein at the 5′ end for visualization (Fig. 2a)^37^. The deadenylation reactions then were analyzed on denaturing gels with single nucleotide resolution of products^45^.

The CCR4a:CAF1 exonuclease heterodimer demonstrated very low deadenylation activity at an equimolar ratio of enzyme heterodimer to the RNA substrate in a time course experiment, consistent with previous studies^37,46^ (Fig. 2b). Efficient deadenylation was observed after the ratio of the enzyme complex was increased to be in excess over the substrate (Supplementary Fig. 2c), up to maximal five-fold (Fig. 2c). The substrate was degraded first to an intermediate product containing, in addition to the 7-mer body, just one or two As (Fig. 2c, 16–24 min) and degradation terminated at the 5′-terminal 5′-UCU-3′ trinucleotide (Fig. 2c, 32–48 min). This is consistent with previous observations that the CCR4a:CAF1 exonuclease module has a preference rather than strict specificity for adenosine^36,46^.

In contrast, CCR4-NOT_FULL_ efficiently degraded the poly(A) tail at an equimolar ratio of complex to the substrate (Fig. 2d vs. b). CCR4-NOT_FULL_ was not only more efficient than the CCR4a:CAF1 heterodimer, but also appeared to be more selective for poly(A), as evident from the stability of the A_1_ and A_0_ products even following extended incubation (Fig. 2c, 24–48 min vs. Fig. 2d, 16–48 min). This suggests that as in yeast the human CCR4-NOT complex exhibits much greater selectivity for poly(A) compared to its constituent exonucleases.

### Two non-enzymatic modules suffice to stimulate CCR4a:CAF1

CCR4-NOT_FULL_ is composed of four distinct structural modules: the NOT10:NOT11, CCR4a:CAF1 exonuclease, CAF40 and NOT modules (Fig. 1a, c). We asked if we could delineate which modules or even individual subunits may contribute directly towards the increased activity and selectivity of deadenylation.

To this end, in addition to CCR4-NOT_FULL,_ we generated a six-subunit complex variant, termed CCR4-NOT_Δ10:11_, in which the NOT10:NOT11 heterodimer was omitted during the reconstitution procedure (Fig. 1b, c). The speed and selectivity of deadenylation by CCR4-NOT_Δ10:11_ appeared very similar to CCR4-NOT_FULL_ (Fig. 2e vs. d). This suggests that under the conditions of the assay and with the same substrate the NOT10:NOT11 heterodimer does not stimulate deadenylation of the CCR4-NOT in presence of the other modules.

Next, we tested another six-subunit complex comprising, in addition to the CCR4a:CAF1 full-length exonucleases, only the C-terminal half of NOT1 (residues 1093-2376), CAF40 (residues 19-285), and the minimal constructs of NOT2 (residues 344-540) and NOT3 (residues 607-753). In these constructs, the extensive N-terminal low-complexity regions of NOT2 and NOT3, as well as the NOT3 coiled-coil region, were removed (i.e., the constructs comprise the functional and structural core of the NOT module; Fig. 1c and Supplementary Fig. 2d). Unlike the larger CCR4-NOT assemblies containing the full-length NOT1 scaffold, all subunits used to reconstitute this complex were produced in bacteria rather than insect cells. Surprisingly, this minimal complex still retained the deadenylation activity almost at the same level as CCR4-NOT_FULL_ (Fig. 2f vs. d) and we termed it CCR4-NOT_MINI_ (Fig. 1c).

Finally, we reconstituted an assembly in which the CAF40 module comprising CAF40 and one domain from the NOT1 scaffold was the only other module present in addition to the exonuclease module comprising CCR4a, CAF1 and a MIF4G domain of NOT1 (the NOT1 construct consists of residues 1093-1607; Fig. 1c and Supplementary Fig. 2d). This four-subunit complex lacking the NOT module, which we termed CCR4-NOT_CORE_, was strikingly less active than CCR4-NOT_MINI_ (Fig. 2g vs. f), but CCR4-NOT_CORE_ was still considerably more active than the CCR4a:CAF1 exonuclease heterodimer (Fig. 2g vs. b) consistent with previous observations^47^.

In summary of the results presented so far, the systematic compositional dissection of human CCR4-NOT revealed that in addition to the CCR4a:CAF1 exonucleases, the non-enzymatic CAF40 and NOT modules are together necessary and sufficient to fully stimulate deadenylation in vitro. In contrast, the NOT10:NOT11 module did not provide substantial additional stimulation. Importantly, in all the CCR4-NOT complexes we compared, the CCR4a:CAF1 exonucleases were purified and incorporated into larger assemblies in an identical manner. This strategy ensured that activity assays were consistently reproducible between different, independently purified batches of complexes.

At 1 µM concentration the NOT10:NOT11 heterodimer is the only subcomplex to bind the 7-mer-A_20_ RNA under the non-equilibrium conditions of electrophoretic mobility shift assays (EMSA) (Fig. 2h). The binding of the CAF40 module was detectable only at 25 µM (Supplementary Fig. 2e). Although we did not observe binding of other modules under these assay conditions, we cannot rule out that these interactions occur in the context of the intact complex. We then performed UV crosslinking experiments that provide further evidence of interactions of the labeled poly(U) RNA with the CCR4-NOT_FULL_, the NOT10:NOT11 heterodimer and, to a lesser degree, also with MBP-tagged CAF40 and the nuclease module (Supplemental Fig. 2f). This indicates that the NOT10:NOT11 and CAF40 modules can directly interact with the RNA, which implies that they may contribute to improved substrate binding. Finally, we show that CCR4-NOT_FULL_ binds preferentially to poly(U) and poly(G) sequences compared to poly(A) and poly(C) under the EMSA conditions (Supplementary Fig. 2g).

### Distinct activities of CCR4a and CAF1 in CCR4-NOT_FULL_

The presence of two seemingly redundant nucleases in the CCR4-NOT complex raises the interesting question as to whether they are indeed redundant or have rather distinct functions. In *S. pombe*, single deactivating mutations of either Ccr4 or Caf1 exonuclease only mildly impaired the activity of the intact Ccr4-Not in vitro and complete deadenylation block was observed only when both catalytic mutants were combined^35^. However, the human CCR4a:CAF1 heterodimer stabilized by the accessory factor BTG2 was reported to be inactive in vitro when either of the exonucleases is mutated^48^.

**Fig. 3:**
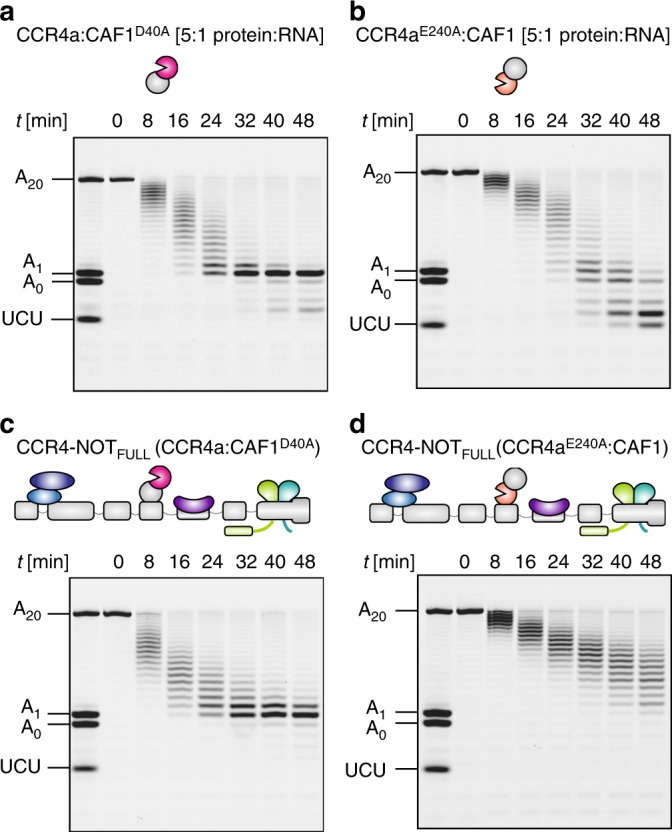
Comparison of deadenylation activities of CAF1 and CCR4a. **a**,**b** Deadenylation assays with the 7-mer-A_20_ RNA substrate (50 nM) and a five-fold molar excess of the CCR4a:CAF1 exonuclease heterodimer (250 nM) containing inactivating mutations in either CAF1 (**a**; D40A mutation) or CCR4a (**b**; E240A mutation), respectively. Both mutations decrease the rate of deadenylation compared to wildtype (Fig. 2c), and the CAF1^D40A^ mutation (**a**) improves the selectivity of the exonuclease heterodimer for adenosine. **c**, **d** Deadenylation assays with equimolar concentrations (50 nM) of 7-mer-A_20_ RNA and CCR4-NOT_FULL_ complexes with the catalytically inactivating mutations in CAF1 (**c**) and CCR4a (**d**), respectively. The CAF1^D40A^ mutation mildly decreases deadenylation activity and leads to increased adenosine specificity compared to wildtype (Fig. 2d), while the CCR4a^E240A^ mutation results in a drastically slower deadenylation reaction. Source data are provided as a Source Data file

To examine the individual contributions of CCR4a and CAF1 we reconstituted heterodimers where one of the enzymes contained an inactivating catalytic mutation: CCR4a^E240A^:CAF1 and CCR4a:CAF1^D40A^ ^35,37,49^. We observed that, in contrast to the reported findings in the presence of BTG2^48^, the catalytic inactivation of either CAF1 or CCR4a had little impact on the deadenylation pattern compared to the wildtype CCR4a:CAF1 (Fig. 3a, b vs. 2c). CCR4a^E240A^:CAF1 was slightly less efficient than CCR4a:CAF1^D40A^ (Fig. 3 b vs. a, 8–24 min). However, there was a clear difference in sequence selectivity in the mutant context with CCR4a:CAF1^D40A^ degrading the substrate to a stable A_1_ product (5′-UCUAAAUA-3′) whereas CCR4a^E240A^:CAF1 efficiently degraded to the 5′-UCU-3′ trinucleotide (Fig. 3a, 32-48 min vs. 3 b, 40–48 min). Thus, CCR4 appears to be more specific for adenosine nucleotides compared to CAF1^37^.

We then asked if CCR4a retains its higher activity and selectivity compared to CAF1 when incorporated into CCR4-NOT_FULL_. Intriguingly, CCR4a:CAF1^D40A^ showed slightly reduced activity in the context of CCR4-NOT_FULL_ compared with wildtype (Fig. 3c, 8–24 min vs. 2d, 8–24 min), but higher sequence selectivity than wildtype CCR4-NOT_FULL_ as the A_1_ product was remarkably stable over the deadenylation time course (Fig. 2d, 24–48 min vs. 3c, 32–48 min). Inactivating CCR4a in the CCR4-NOT_FULL_, however, drastically reduced the deadenylation efficiency compared to wildtype (Fig. 3d vs. 2d) or CCR4-NOT_FULL_ reconstituted with CCR4a:CAF1^D40A^ (Fig. 3d vs. c). Importantly, catalytic inactivation of CCR4a resulted in a much more severe deadenylation defect in the context of CCR4-NOT_FULL_ than in the isolated exonuclease heterodimer (Fig. 3d vs. b) suggesting that the non-enzymatic subunits modulate distinct exonuclease activities.

We have also reconstituted the CCR4-NOT_Δ10:11_ and CCR4-NOT_MINI_ complexes with the CCR4a^E240A^:CAF1 and CCR4a:CAF1^D40A^ exonuclease mutants. The same overall pattern where CCR4a was more active and selective than CAF1 on synthetic substrates was also observed in these complexes (Supplementary Fig. 3a–e). Thus, the distinct properties of CCR4a and CAF1 appear not to alter in response to the non-enzymatic subunit composition. We have also tested a CCR4a^E240A^:CAF1^D40A^ complex where both enzymes were inactivated, and incorporated these inactive exonucleases into CCR4-NOT_FULL_ and CCR4-NOT_MINI_. None of these complexes were active, which suggests our purification procedures were effective in removing any contaminant nuclease activity (Supplementary Fig. 3f–h).

Taken together, these results support a model in which CCR4a rather than CAF1 makes the dominant contribution towards the activity and specificity of deadenylation in the intact human CCR4-NOT complex in vitro and in absence of other factors.

### Peptide motifs compete with RNA for binding to CAF40

The CAF40 subunit is positioned next to CCR4a:CAF1 on the NOT1 scaffold (Fig. 1a) where it serves as a binding platform not only for nucleic acids in vitro (Supplementary Fig. 2e, f)^41^ but also for the α-helical CAF40-binding motifs (CBMs) of metazoan NOT4^50^, as well as the *Drosophila melanogaster* (*Dm*) proteins Bag-of-marbles (Bam; Supplementary Fig. 4a)^20^ and Roquin^13^. To confirm that the CAF40 subunit retains the same binding to the CBMs in the context of the CCR4-NOT_FULL_ and CCR4-NOT_MINI_ complexes, we carried out pulldown assays with MBP-tagged Bam CBM and the Roquin C-terminal region (Supplementary Fig. 4b, c).

**Fig. 4:**
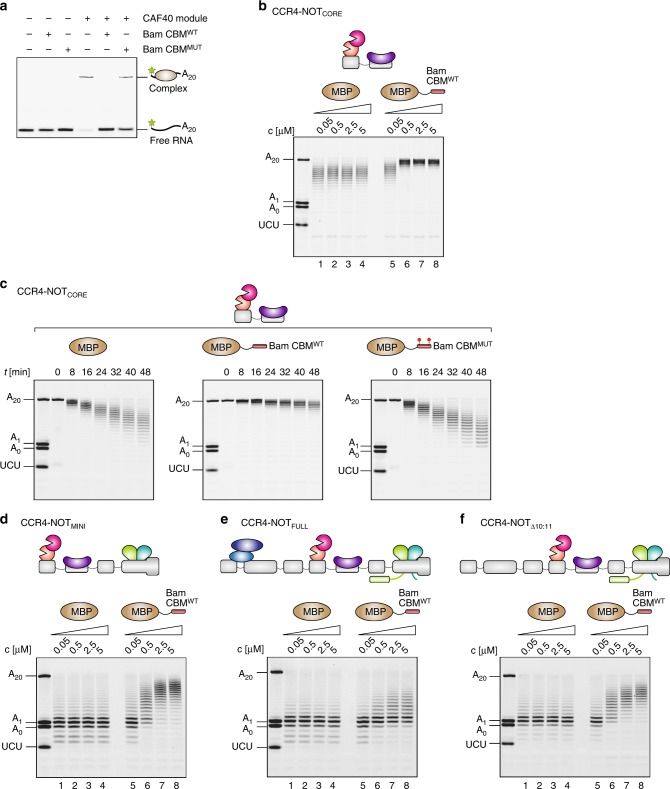
A peptide motif from *Drosophila* Bag-of-marbles inhibits deadenylation. **a** EMSA with the 7-mer-A_20_ RNA and the CAF40 module in the absence or presence of MBP-tagged Bam CBM (wildtype or the double mutant). Only Bam CBM^WT^, but not Bam CBM^MUT^ efficiently competed with RNA for CAF40 binding. **b** Titration experiment with 50 nM of the 7-mer-A_20_ RNA and the CCR4-NOT_CORE_ complex, and the indicated concentrations (0.05–5.0 µM) of either MBP, which served as a negative control, or MBP-tagged Bam CBM^WT^. A 10-fold excess (500 nM) of Bam CBM^WT^ was sufficient to almost completely inhibit deadenylation. The reactions were stopped after 32 min. **c** Time course assay with the 7-mer-A_20_ RNA and the CCR4-NOT_CORE_ complex in equimolar ratio (50 nM). In addition, a 50-fold molar excess (2.5 µM) of either MBP, which served as a negative control, or MBP-tagged Bam CBM^WT^ or Bam CBM^MUT^ (containing L17E and M24E mutations) were included. **d**–**f** Titration experiments with 50 nM of the 7-mer-A_20_ RNA and the CCR4-NOT_MINI_ (**d**), CCR4-NOT_FULL_ (**e**) or CCR4-NOT_Δ10:11_ (**f**) complexes, respectively, and the indicated concentrations (0.05–5.0 µM) of either MBP, which served as a negative control, or MBP-tagged Bam CBM^WT^. The reactions were stopped after 32 min. Source data are provided as a Source Data file

Protein–protein and protein–RNA interactions on CAF40 are shared via a common surface^13,20,41,50^, and consequently, they are likely to be mutually exclusive. To test this, we initially set out to test competition between RNA and protein binding by CAF40. We selected the CBM of Bam as a candidate deadenylation antagonist due to its nanomolar affinity for CAF40 and structurally characterized binding mode^20^ (Supplementary Fig. 4d). Indeed, MBP-tagged Bam CBM^WT^ efficiently prevented CAF40 from RNA binding in an EMSA (Fig. 4a). In contrast, a Bam CBM containing amino acid substitutions L17E and M24E designed to disrupt binding to CAF40 (Supplementary Fig. 4d)^20^, which we termed Bam CBM^MUT^, had almost no effect on RNA binding by CAF40 (Fig. 4a).

Then, we devised an assay to investigate the impact of Bam CBM^WT^ on the CAF40-mediated stimulation of deadenylation. We chose to initially test it on CCR4-NOT_CORE_ as this complex is more active than the exonucleases and the CAF40 module is the only non-enzymatic module present.

The deadenylation activity of CCR4-NOT_CORE_ was strongly reduced when we titrated increasing amounts of MBP-tagged Bam CBM^WT^ (Fig. 4b, lanes 5-8) compared to the MBP control (Fig. 4b, lanes 1-4). Strong inhibition was also evident in a time course assay in the presence of an almost saturating concentration of the Bam CBM^WT^, equivalent to 50-fold molar excess over CAF40 (Fig. 4c, left and middle panels). Indeed, the activity of CCR4-NOT_CORE_ with this concentration of Bam CBM^WT^ was reduced almost to the level of the CCR4a:CAF1 heterodimer alone (Fig. 4c, middle panel vs. Fig. 2b). The activity of the exonuclease heterodimer alone was not inhibited by Bam CBM^WT^ (Supplementary Fig. 4e). Importantly, the activity of CCR4-NOT_CORE_ was not affected when we added an equivalent molar excess of Bam CBM^MUT^ or with the MBP control (Fig. 4c, left and right panels). This suggests that deadenylation inhibition is directly correlated to high-affinity binding of this linear peptide motif to CAF40.

### The NOT10:NOT11 module compensates for CAF40 unavailability

To assess if the Bam CBM can inhibit if the other stimulatory sites of the CCR4-NOT complex are available, we tested its effect on the deadenylation ability of our larger assemblies. Initially, we observed that CCR4-NOT_MINI_ was also progressively inhibited by increasing amounts of Bam CBM (Fig. 4d), but not as efficiently as CCR4-NOT_CORE_ (Fig. 4b), suggesting that the NOT module can partially compensate for the loss of deadenylation stimulation by CAF40. CCR4-NOT_FULL_ was even more resistant to inhibition by Bam CBM^WT^. Unlike with CCR4-NOT_MINI_, the activity of CCR4-NOT_FULL_ was only slightly affected during titration with Bam CBM^WT^ (Fig. 4e). This difference between the two complexes suggests that the NOT10:NOT11 module is primarily responsible for the resistance of CCR4-NOT_FULL_ against the inhibition by the Bam CBM. This was confirmed by the observation that in presence of Bam CBM^WT^, the deadenylation activity of CCR4-NOT_Δ10:11_ was significantly inhibited compared to the CCR4-NOT_FULL_ and rather resembled the situation in CCR4-NOT_MINI_ (Fig. 4f).

Given that CAF40 is conserved and in an assumed spatial proximity to the exonucleases, we suggest that CAF40 provides the principal stimulatory surface. If this is blocked by a linear motif from an interacting partner, then the NOT and NOT10:NOT11 modules can potentially compensate.

### Multiple CCR4-NOT interactors can inhibit deadenylation

**Fig. 5:**
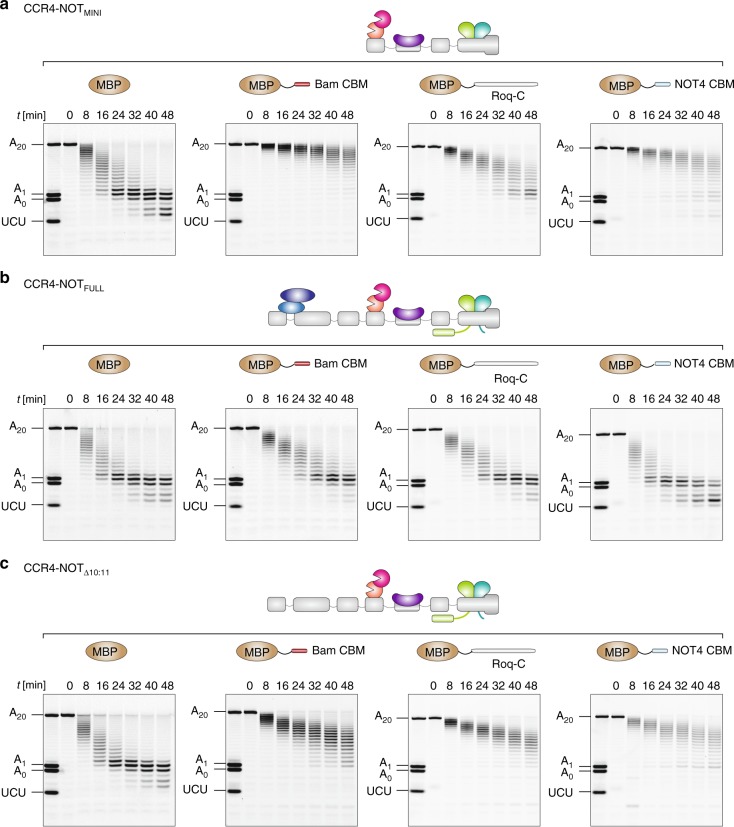
Motifs from multiple CCR4-NOT recruitment factors inhibit deadenylation. **a**–**c** Deadenylation assays with equimolar concentrations (50 nM) of the 7-mer-A_20_ substrate and the CCR4-NOT_MINI_ (**a**), CCR4-NOT_FULL_ (**b**), or CCR4-NOT_Δ10:11_ (**c**) complexes, respectively. 2.5 µM of either MBP (as a negative control) or MBP-tagged Bam CBM^WT^, Roquin-C or NOT4 CBM, respectively, were tested with the reconstituted complexes. All three motifs inhibited the deadenylation reaction of all three complexes but the extent of the inhibition was variable. The CCR4-NOT complexes lacking the NOT10:NOT11 heterodimer were strikingly more sensitive to the inhibition by the peptide motifs. Source data are provided as a Source Data file

To determine if the deadenylation inhibition via the CBM is unique to Bam or whether CBMs from other RNA-binding recruitment factors can also impact on CCR4-NOT activity, we tested an extended C-terminal fragment of human Roquin1 (Roq-C), which binds directly to the CAF40 subunit and the NOT module (Supplementary Fig. 4f)^13,20^, as well as a CBM from the C-terminus of NOT4 which binds to the same surface as the Bam CBM, but in a structurally unrelated manner (Supplementary Fig. 4g)^50^. In a series of deadenylation time courses, where we systematically tested the effect of all three MBP-tagged peptides (Bam CBM^WT^, Roq-C, and NOT4 CBM) on CCR4-NOT_MINI_ (Fig. 5a), CCR4-NOT_FULL_ (Fig. 5b) and CCR4-NOT_Δ10:11_ (Fig. 5c), we observed that peptide motifs from all three factors inhibit CCR4-NOT-mediated deadenylation in vitro. However, the extent of inhibition varied among the complexes. The complexes lacking the NOT10:NOT11 heterodimer, CCR4-NOT_Δ10:11_ and CCR4-NOT_MINI_, were strikingly more sensitive to the inhibition by the peptides compared to the CCR4-NOT_FULL_. Taken together, these results show that multiple interaction partners of the CCR4-NOT complex can repress deadenylation via direct binding to CAF40 and the NOT10:NOT11 module compensates by restoring deadenylation stimulation.

### 3′-UTR length but not composition affects deadenylation

**Fig. 6:**
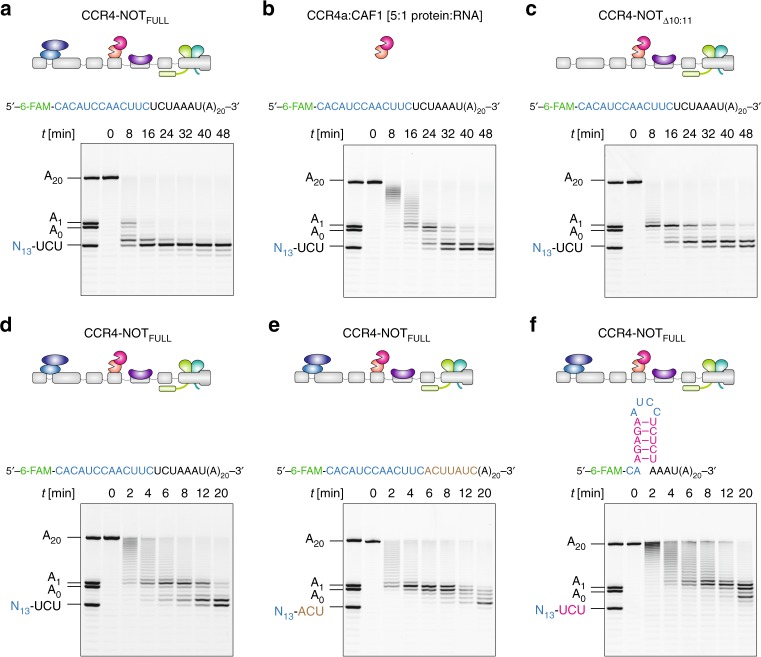
The length of the segment preceding the poly(A) tail but not its sequence impacts on deadenylation. **a** Deadenylation assay with the CCR4-NOT_FULL_ complex and the 13+7-mer-A_20_ RNA substrate in equimolar ratio (50 nM). This substrate contains an additional unstructured 13-mer segment 5′ to the 7-mer-A_20_ RNA. The deadenylation of this substrate is markedly enhanced compared to the 7-mer-A_20_ substrate (Fig. 2d) and did not proceed beyond the first two consecutive non-A nucleotides. **b** Deadenylation assay with the same 13+7-mer-A_20_ substrate (50 nM) and a five-fold molar excess of the CCR4a:CAF1 exonuclease heterodimer (250 nM). The rate of deadenylation does not differ to that with the 7-mer-A_20_ substrate indicating that the exonuclease heterodimer does not discriminate between these substrates (Fig. 2c). **c** Deadenylation assay with the CCR4-NOT_Δ10:11_ complex and the 13+7-mer-A_20_ RNA substrate in equimolar ratio (50 nM). **d** Deadenylation assay with the CCR4-NOT_FULL_ complex and the 13+7-mer-A_20_ substrate (both 50 nM) with higher resolution of the early time points compared to **a**. **e** Deadenylation assay with the CCR4-NOT_FULL_ complex and a 20-mer-A_20_ substrate where the seven nucleotides immediately preceding the poly(A) tail were different from the 13+7-mer-A_20_ substrate in equimolar ratio (50 nM). **f** Deadenylation assay with the CCR4-NOT_FULL_ complex and the 13-SL-7-mer-A_20_ substrate containing a short stem-loop structure within the 20-nucleotide region upstream of A_20_ in equimolar ratio (50 nM) (see also Supplementary Table 4). Source data are provided as a Source Data file

The interactions of several stimulatory modules with RNA suggested that longer substrates may engage several stimulatory surfaces at the same time. This would stabilize the enzyme-substrate complex and lead to more efficient deadenylation. To test this, we performed deadenylation assays using a substrate with an extra 13 randomly chosen nucleotides, which are predicted to be unstructured, 5′ of the 7-mer-A_20_ (Supplementary Table 4). This RNA substrate interacts stably with the CCR4-NOT_FULL_ complex, as well as the NOT10:NOT11 heterodimer under the EMSA conditions (Supplementary Fig. 5a), and at higher concentrations with the CAF40 module (Supplementary Fig. 5b), similar to the shorter 7-mer-A_20_ substrate (Fig. 2h and Supplementary Fig. 2e). We observed an enhanced deadenylation activity of CCR4-NOT_FULL_ on this 13 + 7-mer-A_20_ substrate compared to 7-mer-A_20_ (Fig. 6a, 16 min vs. 2d, 48 min, and Fig. 6d). This result is in contrast to the reported situation in fission yeast where the length upstream of the poly(A) did not significantly influence activity^35^. At the same time, the CCR4-NOT complex did not degrade this substrate beyond the first two consecutive non-A nucleotides, consistent with the notion that the high level of selectivity for adenosine is not influenced significantly by the overall length of the substrate. Strikingly, we did not observe a similar enhancement with the longer substrate on the activity of the CCR4a:CAF1 heterodimer (Fig. 6b vs. 2c). Furthermore, the absence of the NOT10:NOT11 module reduced the deadenylation activity on the longer substrate only slightly (Fig. 6c vs. a, and Supplementary Fig. 5c).

To assess whether the sequence of the region preceding the poly(A) influences deadenylation by CCR4-NOT_FULL_, we performed deadenylation assays using four different substrates, each with 20 nucleotides upstream of A_20_ (Supplementary Table 4). Surprisingly, the differences between three substrates with unstructured 5′ regions were not very pronounced (Fig. 6d, e, Supplementary Fig. 5d), suggesting that the sequence composition of 3′-UTR does not significantly influence deadenylation. Introduction of a short stem-loop structure led to an initial decrease in the rate of deadenylation but the reaction still proceeded to completion (Fig. 6f vs. d, e). This suggests that the presence of secondary structure in the 3′-UTR is not a critical determinant of deadenylation efficiency.

### The CCR4-NOT complex is a conformationally flexible assembly

We used negative stain electron microscopy (EM) to characterize the particle shape and dimensions of CCR4-NOT_FULL_. This revealed many particles of different size and shape (Supplementary Fig. 6a). Their size heterogeneity and globular appearance suggested they were isolated subunits or subcomplexes. Localized particle clustering further indicated that CCR4-NOT_FULL_ disassembles when stained with acidic uranyl formate. To stabilize complexes for EM, we employed mild crosslinking using glutaraldehyde during sucrose density gradient centrifugation^51^. This markedly reduced nonspecific aggregation and complex disassembly resulting in a more homogeneous particle size distribution (Supplementary Fig. 6b). The average length of a particle was approximately 22–24 nm in the longest direction, consistent with the reported dimensions observed by negative stain EM for the yeast Ccr4-Not complexes^52,53^. However, particle shape and appearance indicates that CCR4-NOT_FULL_ adopts not one single conformation but rather a continuum of many different conformations, which means that many of the subunits and modules may be able to locate in close spatial proximity.

## Discussion

The human CCR4-NOT complex was recently shown to be the principal mediator of deadenylation^7^. Here, we describe the production of a fully recombinant human CCR4-NOT complex comprising all eight subunits, with only the NOT11 subunit containing a significant truncation of the unstructured N-terminal region. Through a modular reconstitution approach, we systematically characterized the contribution of non-enzymatic modules towards deadenylation.

We observed that the intact complex is strikingly more active compared to the isolated CCR4a:CAF1 heterodimer, consistent with observations in fission yeast^35^. The CCR4-NOT complex is also much more sequence-specific as evident by stalling following efficient shortening of the substrate poly(A) tail. Previously it was shown that recombinant CCR4b is significantly more selective towards polyadenosine in synthetic substrates compared to CAF1^36,37,54^. Our studies with mutant nucleases incorporated into the CCR4-NOT indicated that CCR4a is principally responsible for sequence selectivity and activity, whereas CAF1 was rather inactive in three distinct compositional variants of the CCR4-NOT under the assay conditions. This contrasts markedly with the fission yeast complex where Caf1 and Ccr4 catalytic mutants had almost identical deadenylation profiles^35^. CCR4a and CAF1 were reported to be functionally distinct depending on whether the poly(A) tail is coated with the poly(A)-binding protein (PABPC1)^7,35^. Our results indicate that the functional distinction between CCR4a and CAF1 exists also in the absence of PABPC1.

The activity of the fission yeast Ccr4-Not was shown to depend on the sequence and the secondary structure content of the 3′-UTR but not its length. It was thus proposed that the Ccr4-Not has an intrinsic ability to recognize and respond to the sequence and/or structure context upstream of the poly(A) tail^35^. Human CCR4-NOT, in contrast, showed a strong dependence on the length of the 3′-UTR segment. Since the exonuclease module does not bind the substrate well, the purpose of multiple independent RNA-binding sites on spatially distinct modules may be to strengthen the interaction of the complex with its substrate via avidity effects and thereby improve deadenylation efficiency. Furthermore, our data indicate that the human CCR4-NOT is not selective at the sequence level for 3′-UTR and deadenylation activity is not significantly impacted by the secondary structure. This is consistent with a view that the CCR4-NOT may be more reliant on extrinsic factors for 3′-UTR discrimination than the yeast complex. This may increase the potential for a very fine, transcript-specific regulation of gene expression necessary in multicellular organisms.

We observed that the CCR4-NOT-mediated deadenylation is only efficiently terminated once a stretch of more than two non-adenosine residues is encountered by the complex. The CCR4-NOT thus has an intrinsic preference for poly(A), which is enhanced by the non-enzymatic subunits and is independent of the sequence context of the 3′-UTR. Collectively, our findings support a view of the human CCR4-NOT where non-enzymatic subunits serve not only to enhance the exonuclease activity but to exquisitely tune it for the purpose of poly(A) shortening and to minimize nonspecific RNA degradation.

We observed that at least three independent sites on the human CCR4-NOT act in concert to stimulate deadenylation. The CAF40 subunit binds RNA directly via a conserved surface and is sufficient to strongly stimulate activity. CAF40 is proximal to the exonuclease module, which does not bind substrates stably, and thus CAF40 may stimulate deadenylation through improved substrate binding or through optimized orientation of the RNA for degradation.

In addition to the CAF40 module, the structural core of the NOT module, consisting of the NOT2 and NOT3 subunits, as well as the C-terminal domain of NOT1, is necessary and sufficient to achieve full stimulation in vitro. In our binding assays, the human NOT module does not bind RNA directly unlike the yeast NOT module, which was shown to bind nucleic acid in vitro^44^. The NOT module is critical for the stability of CCR4-NOT in *Drosophila* S2 cells^39^ and we suggest this role is conserved in the human complex thus aiding its activity but the precise mechanistic contribution remains unclear at present.

The NOT10:NOT11 module provides the third potent stimulatory site. The module is conserved in many eukaryotes, with the exception of unicellular yeasts, but its function remains unknown although it appears to serve an important function in mammals as *Cnot10* deficiency in mice causes embryonic lethality^55^. It binds RNA directly and with the highest relative affinity of all modules tested in this study (Fig. 2h). Thus, we suggest that NOT10:NOT11 stimulates deadenylation through direct stabilization of the substrate RNA but only under distinct conditions where the principal stimulatory CAF40 surface may not be available for RNA binding.

**Fig. 7:**
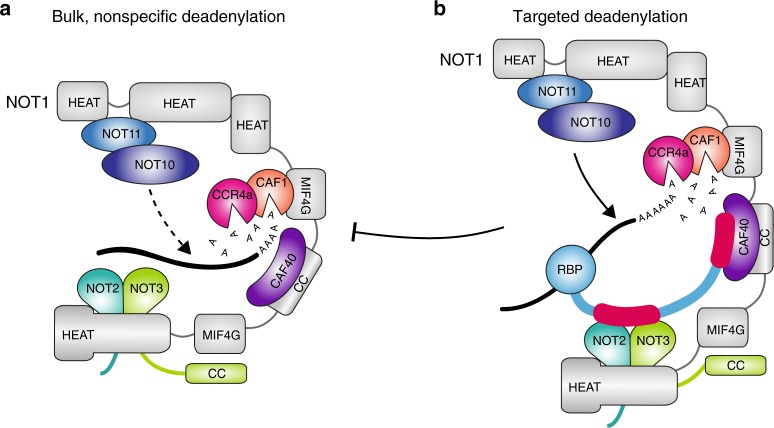
A proposed mechanistic model for the switch from bulk to targeted deadenylation. **a** The CCR4-NOT complex possesses intrinsic affinity towards nucleic acids which allows it to bind to and deadenylate bulk mRNAs in a manner independent of the sequence of the RNA. In addition to the active sites of the nucleases, also the CAF40 module, the NOT module, and the NOT10:NOT11 module can directly interact with the substrate and contribute to a different extent to the stimulation of deadenylation, with CAF40 being the dominant stimulatory site under the conditions of our in vitro assays. **b** Upon stimulation, RNA-binding proteins (RBPs) can recruit the CCR4-NOT complex to specific transcripts via direct protein-protein interactions and trigger the preferred deadenylation of those transcripts. In addition, RBPs carrying a CAF40-binding motif can mask the RNA-binding site on CAF40, thereby gaining further competitive advantage for deadenylation of their bound mRNA over other, nonspecific transcripts in the cytoplasmic mRNA pool

Taken together, we propose a mechanistic model in which RNA-binding surfaces on distinct modules cooperate to enhance interaction with the substrate to elicit efficient deadenylation. Such interactions may also exploit the inherent structural flexibility of CCR4-NOT to position the mRNA substrate in an optimal conformation (Fig. 7a).

Linear motifs from several mRNA-binding proteins can bind to the highly conserved nucleic acid binding site on CAF40 to compete with RNA binding and interfere with deadenylation stimulation. The long-standing notion of targeted deadenylation by the CCR4-NOT is a unidirectional one in which CCR4-NOT acts on bulk mRNA and is repurposed toward specific mRNA targets by protein factors. Our biochemical data suggests that blocking an RNA-binding surface on CAF40 by recruitment factors may reduce nonspecific substrate binding and bulk deadenylation but this model remains to be tested in vivo (Fig. 7b). Our findings indicate that there is some RNA-binding redundancy in the CCR4-NOT modules. Recruitment factors such as GW182^33^, TTP^56^, and Roquin^13^ bind to several CCR4-NOT subunits through extended motifs. Multiple recruitment and regulatory events could perhaps occur simultaneously on the multisubunit CCR4-NOT and access to specific stimulatory sites may be regulated in specific contexts. An important future goal is to understand how the conformational dynamics of the CCR4-NOT complex are influenced through combinatorial control from multiple regulatory inputs in response to stimuli to ultimately redirect the gene expression program of specific targets.

In conclusion, our compositional dissection of the human CCR4-NOT reveals that many parts of this multisubunit complex act together and intrinsically modulate each other to coordinate the shortening of poly(A) tails. We propose a mechanistic model of allosteric and cooperative stimulation of the exonucleases where several non-enzymatic modules mediate interactions with the RNA substrate. Means of producing the recombinant and biochemically tractable intact human CCR4-NOT will facilitate the future studies of targeted deadenylation, translational repression, and co-translational decay.

## Methods

### DNA constructs

For expression of the NOT1:NOT2:NOT3:CAF40 complex (Supplementary Fig. 1a and 7a), full-length NOT1 was amplified from a human cDNA library, and sequences encoding an N-terminal His_10_ (fused to NOT1 via a TEV protease site) and two consecutive C-terminal StrepII tags were added during PCR. The DNA sequence encoding full-length CAF40 was amplified from the same cDNA library. Then, the NOT1 and CAF40 PCR products were fused together, each with a downstream SV40 poly(A) signal and an upstream polyhedrin promoter, into a single cassette by overlap extension PCR, and this cassette was inserted between the SalI and ScaI restriction sites into the pACEBac1 vector backbone (Supplementary Fig. 7b). The oligonucleotide primers used in this study are listed in Supplementary Table 5.

cDNA encoding full-length NOT2 was also amplified from the cDNA library, and the cleaved PCR product inserted between the XhoI and NcoI restriction sites of the pIDK plasmid. Amplified cDNA encoding full-length NOT3 was inserted in the KpnI restriction site of the pIDK plasmid. Following this, the NOT2 cassette including the upstream polyhedrin promoter and the downstream SV40 poly(A) signal was amplified from the pIDK-NOT2 plasmid and inserted into the ClaI restriction site of pIDK-NOT3 to create the pIDK-NOT2-NOT3 plasmid (Supplementary Fig. 7b).

Finally, the pACEBAC-NOT1-CAF40-NOT2-NOT3 bacmid was created in vitro by Cre-Lox recombination using Cre recombinase (New England Biolabs) with 200 fmol of pACEBac-NOT1-NOT9 and 300 fmol pIDK-NOT2-NOT3 in the reaction.

cDNA encoding full-length CCR4a was inserted into the pMCSG19c plasmid vector^57^ between KpnI and BamHI restriction sites (Supplementary Fig. 7c). In this vector an insert is fused in-frame with a gene encoding maltose-binding protein (MBP) with an adjacent TVMV protease cleavage site. Low-level production of encoded TVMV protease from the same plasmid cleaves the fused MBP in vivo resulting in a CCR4a protein construct with an N-terminal His_6_-tag followed by the TEV protease recognition site.

CAF1 cDNA fused to an N-terminal TEV site was inserted between the BamHI and HindIII restriction sites of the pET28b plasmid (Merck). To improve the stability of CAF1, a SUMO3 tag with an N-terminal His_6_ tag was inserted between NdeI and BamHI restriction sites, resulting in a SUMO-His_6_-CAF1 fusion construct cleavable by TEV protease (Supplementary Fig. 7c).

The NOT10:NOT11 heterodimer for reconstitution was co-expressed from two plasmids: cDNA encoding NOT10 (residues 25-707) was inserted between the XhoI and BamHI restriction sites of the pnYC-pM plasmid^58^, resulting in an MBP-tagged fusion construct cleavable by HRV-3C protease (Supplementary Fig. 7d). The NOT11 C-terminal construct (residues 257-498) was inserted between the same restriction sites of the pnEA-vH plasmid^58^, and the resulting C-terminally His_6_-tagged construct was cleavable by TEV protease (Supplementary Fig. 7d).

For expression of the tetramer which serves as a scaffold of the CCR4-NOT_MINI_ complex (Supplementary Fig. 8a), two plasmids were used. MBP-tagged NOT1 (residues 1093-2376) encoded on a plasmid of the pnYC backbone which has been described before^13^ (Supplementary Fig. 8b). A tricistronic plasmid encoding for MBP-tagged NOT2 (residues 344-540), His_6_-tagged NOT3 (residues 607-753) and His_6_-tagged CAF40 (residues 19-285) was constructed based on the pnEA architecture^58^. All tags were cleavable by HRV-3C protease apart from the His_6_ tag fused to NOT3 which was non-cleavable (Supplementary Fig. 8b).

Two plasmids were used for the expression and purification of the NOT1:CAF40 complex serving as the base for reconstitution of the CCR4-NOT_CORE_ complex (Supplementary Fig. 8c). cDNA encoding for NOT1 (residues 1093-1607) was inserted between the XhoI and BamHI restriction sites of the pnYC-pM plasmid backbone. CAF40-encoding cDNA (residues 19-285) was inserted between the XhoI and BamHI restriction sites of a pnEA plasmid which does not encode any affinity tags (Supplementary Fig. 8d).

For expression of MBP-tagged CAF40, cDNA encoding CAF40 residues 18-293 was inserted between the XhoI and BamHI restriction sites of the pnEA-pM plasmid^58^.

The DNA constructs for the production of the individual CCR4-NOT modules (the CAF40 module comprising NOT1 residues 1351-1588 and CAF40 residues 19-285, and the NOT module core comprised of NOT1 residues 1833-2361, NOT2 residues 350-540 and NOT3 residues 607-748), MBP-tagged *Drosophila* Bam CBM and MBP-tagged *Hs* Roquin1 CBM have been described before, as well as the detailed description of expression and purification of these constructs^11,13,20,33^.

Site-directed mutagenesis was carried out according to modified QuickChange^TM^ protocol^59^. All the plasmid constructs and mutants used in this study were confirmed by DNA sequencing and are listed in Supplementary Table 2.

### Baculovirus production

Recombined multi-plasmid vectors were transformed into chemically competent DH10EmbacY cells (a kind gift from Imre Berger). Positive baculovirus genome integrands were selected using blue/white screening on LB-agar plates containing IPTG and Bluo-Gal (Gold Biotechnology) with 25 µg/ml kanamycin, 10 µg/ml tetracycline, 34 µg/ml chloramphenicol, and 10 µg/ml gentamicin. Two white colonies were used to inoculate 5 ml LB cultures with all four antibiotics at the same concentrations and cultured at 37 °C overnight. Bacmid DNA was purified by alkaline lysis with buffers P1, P2, and N3 (Qiagen). Following isopropanol precipitation, pellets were washed with ice-cold 70% (v/v) ethanol and centrifuged at 4 °C before being resuspended in sterile, milliQ-grade water.

Sterile 1–5 µg bacmid DNA was transfected into a standard sterile 6-well plate (Greiner) with technical replicates of 1.0 × 10^6^ adherent *Sf*21 cells (a kind gift from Imre Berger) using Fugene HD reagent (Promega). The supernatant containing the initial V_0_ low-titer baculovirus was collected once at least 50% of cells were fluorescent due to the expression of the YFP marker. Suspension cultures at 1.0 × 10^6^ cells/ml were infected with approximately 10% (v/v) V_0_ virus stock. A working V_1_ stock of baculovirus was collected 72 h after infection. Cells were cultured in Sf900II serum-free medium (Thermo Fisher Scientific) without any supplements.

### Purification of the CCR4a:CAF1 exonuclease heterodimer

Full-length CCR4a and CAF1 were co-expressed in *E. coli* BL21(DE3) Star cells (Thermo Fisher Scientific) in LB medium at 20 °C as fusion proteins carrying N-terminal His_6_ and His_6_-SUMO tags, respectively. Cells were lysed using an Emulsiflex-C3 homogenizer (Avestin) in a buffer containing 50 mM potassium phosphate pH 7.5, 300 mM NaCl and 25 mM imidazole supplemented with complete EDTA-free protease inhibitors, 5 µg/ml DNaseI and 1 mg/ml lysozyme. The proteins were isolated from the cleared lysate by binding to a Nickel-charged HiTrap IMAC column (GE Healthcare) and eluted from the column by a linear gradient to the same buffer supplemented with 500 mM imidazole. Both tags were then cleaved off overnight by incubation with recombinant TEV protease while dialyzing against a buffer containing 30 mM HEPES/NaOH pH 7.5, 300 mM NaCl and 2 mM DTT. Then, the CCR4a:CAF1 heterodimer was eluted on a Superdex 200 26/600 size exclusion chromatography column (GE Healthcare) equilibrated in a buffer containing 10 mM HEPES/NaOH pH 7.5, 300 mM NaCl and 2 mM DTT. Finally, the NaCl concentration was diluted to 75 mM, and the complex bound to a Source 15Q column (GE Healthcare) was subsequently eluted by a linear gradient to buffer containing 10 mM HEPES/NaOH pH 7.5, 1000 mM NaCl and 2 mM DTT. The eluted CCR4a:CAF1 complex was concentrated to 6-7 mg/ml, flash-frozen and stored at −80 °C. Mutant variants of CCR4a:CAF1 were purified in the same way as the wild-type heterodimer. The CCR4a:CAF1 dimer purified following this scheme was used for reconstitution of all larger complexes (CCR4-NOT_FULL_, CCR4-NOT_Δ10:11_, CCR4-NOT_MINI_, and CCR4-NOT_CORE_).

### Purification of the NOT10:NOT11 heterodimer

Both proteins were co-expressed in *E. coli* BL21(DE3) Star cells in LB medium at 20 °C as fusion proteins carrying C-terminal His_6_ (NOT11, residues 257-498) and N-terminal MBP (NOT10, residues 25-707) tags, respectively. Cells were lysed using an Emulsiflex-C3 homogenizer (Avestin) in a buffer containing 50 mM HEPES/NaOH pH 7.5, 300 mM NaCl and 25 mM imidazole supplemented with complete EDTA-free protease inhibitors, 5 µg/ml DNaseI and 1 mg/ml lysozyme. The proteins were isolated from the cleared lysate by using a nickel-charged HiTrap IMAC column (GE Healthcare) and eluted by a linear gradient to a buffer containing 50 mM HEPES/NaOH pH 7.5, 300 mM NaCl and 500 mM imidazole. Subsequently, the tags were cleaved off overnight by incubation with recombinant HRV-3C and TEV proteases, while dialyzing against a buffer containing 50 mM HEPES/NaOH pH 7.5, 300 mM NaCl, 10% (v/v) glycerol and 2 mM DTT. Then, the NOT10:NOT11 heterodimer was eluted on a Superdex 200 26/600 size exclusion chromatography column (GE Healthcare) equilibrated in a buffer containing 10 mM HEPES/NaOH pH 7.5, 300 mM NaCl, 10% (v/v) glycerol and 2 mM DTT. The NOT10:NOT11 heterodimer was concentrated to 6–7 mg/ml, flash-frozen and stored at −80 °C.

### MBP-CAF40 purification

CAF40 (residues 18-293) was expressed in *E. coli* BL21(DE3) Star cells in LB medium at 20 °C with an N-terminal MBP tag. Cells were lysed using an Emulsiflex-C3 homogenizer (Avestin) in a buffer containing 50 mM HEPES/NaOH pH 7.5, 500 mM NaCl, 10% (v/v) glycerol, and 2 mM DTT. The complex was isolated from the crude lysate using amylose resin (New England Biolabs) and eluted with lysis buffer supplemented with 30 mM D-(+)-maltose. The eluted protein was diluted to reach a final concentration of 100 mM NaCl. This was then applied on a 5 ml heparin column (GE Healthcare) and eluted with a linear gradient to 1 M NaCl. This was followed by size exclusion chromatography on a Superdex 200 26/60 column (GE Healthcare) equilibrated in a buffer containing 10 mM HEPES/NaOH pH 7.5, 200 mM NaCl, 10% (v/v) glycerol, and 2 mM DTT. The protein was concentrated to approximately 25 mg/ml.

### Reconstitution of CCR4-NOT_FULL_ and CCR4-NOT_Δ10:11_ complexes

*Sf*21 insect cells were grown at 27 °C to a density of 2.0 × 10^6^ cells/ml in Sf900II medium (Thermo Fisher Scientific) and infected with recombinant NOT1:NOT2:NOT3:CAF40 baculovirus stock (1:100 v/v). The cells were harvested by centrifugation 48 h after they stopped dividing and pellets stored at −80 °C.

Pellets of insect cells expressing the NOT1:NOT2:NOT3:CAF40 tetramer were thawed, resuspended in lysis buffer (50 mM HEPES/NaOH pH 7.5, 500 mM NaCl, 10 mM potassium phosphate pH 7.5, 50 mM imidazole, 5 µg/ml DNAseI) and lysed by sonication (Branson Sonifier; level 2; 20% output; 1 min). The lysate was cleared by centrifugation (15 min at 12,500 × *g*) and filtered through 0.45 µm and 0.22 µm syringe-driven filters (Millipore). The cleared and filtered lysate was applied to a nickel-charged 1 ml HiTrap IMAC column (GE Healthcare) and the bound complex eluted by a linear gradient to a buffer containing 50 mM HEPES/NaOH pH 7.5, 300 mM NaCl, 10 mM potassium phosphate pH 7.5 and 500 mM imidazole.

For reconstitution of CCR4-NOT_FULL_, the NOT1:NOT2:NOT3:CAF40 subcomplex was mixed in a 1:2:2 molar ratio with CCR4a:CAF1 (wildtype or mutants) and NOT10:NOT11 complexes. The mixture was then incubated on ice for 2 h to assure the association of the subcomplexes with each other, and then the assembled complex was separated from the excess of CCR4a:CAF1 and NOT10:NOT11 heterodimers by size exclusion chromatography on a Sephacryl HR-300 26/60 column (GE Healthcare) equilibrated in a buffer containing 10 mM HEPES/NaOH pH 7.5, 200 mM NaCl, and 2 mM DTT. The complex was concentrated to approximately 1 mg/ml, and for deadenylation assays, it was subsequently aliquoted, flash-frozen and stored at −80 °C.

Procedures were identical for the reconstitution of the CCR4-NOT_Δ10:11_ complex with the difference that the NOT10:NOT11 heterodimer was omitted from the mixture of subcomplexes.

### Reconstitution of the CCR4-NOT_MINI_ complex

MBP-tagged NOT1 (residues 1093-2371), MBP-tagged NOT2 (residues 344-540), His_6_-tagged NOT3 (residues 607-753) and His_6_-tagged CAF40 (residues 19-285) were co-expressed in *E. coli* BL21(DE3)Star cells in LB medium at 20 °C. Cells were lysed using an Emulsiflex-C3 homogenizer (Avestin) in a buffer containing 50 mM potassium phosphate pH 7.5 and 300 mM NaCl supplemented with complete EDTA-free protease inhibitors, 5 µg/ml DNaseI and 1 mg/ml lysozyme. The complex was isolated from the crude lysate using amylose resin (New England Biolabs) and eluted with lysis buffer supplemented with 25 mM D-(+)-maltose. The tags were removed by cleavage with HRV-3C protease.

Subsequently, a two-fold molar excess of purified CCR4a:CAF1 heterodimer (wildtype or mutants) was added and the mixture incubated for two hours. Finally, the assembled complex was separated from the affinity tags and the excess of CCR4a:CAF1 by size exclusion chromatography on a Superdex 200 26/60 column (GE Healthcare) equilibrated in a buffer containing 20 mM HEPES/NaOH pH 7.5, 300 mM NaCl and 2 mM DTT. The complex was concentrated to approximately 1 mg/ml.

### Reconstitution of the CCR4-NOT_CORE_ complex

The heterodimer of NOT1 (residues 1093-1607) and CAF40 (residues 19-285) was co-expressed in *E. coli* BL21(DE3) Star cells in LB medium at 20 °C with N-terminal MBP and His_6_ tags, respectively. Cells were lysed using an Emulsiflex-C3 homogenizer (Avestin) in a buffer containing 50 mM potassium phosphate pH 7.5 and 300 mM NaCl supplemented with complete EDTA-free protease inhibitors, 5 µg/ml DNaseI and 1 mg/ml lysozyme. The complex was isolated from the crude lysate using amylose resin (New England Biolabs) and eluted with lysis buffer supplemented with 25 mM D-(+)-maltose. The tags were removed by cleavage with HRV-3C protease.

Then, a two-fold molar excess of purified CCR4a:CAF1 heterodimer was added and the mixture incubated for two hours. Finally, the assembled complex was separated from the affinity tags and the excess of CCR4a:CAF1 by size exclusion chromatography on a Superdex 200 26/60 column (GE Healthcare) equilibrated in a buffer containing 20 mM HEPES/NaOH pH 7.5, 300 mM NaCl and 2 mM DTT. The complex was concentrated to approximately 1 mg/ml.

The residue counts and molecular weights of all recombinant complexes and their subunits are listed in Supplementary Table 3.

### Size exclusion chromatography with light scattering

The purified CCR4-NOT_FULL_ complex (40 µl at 0.9 mg/ml) was applied to a Superose 6 5/150 column (GE Healthcare) equilibrated in a buffer containing 10 mM HEPES/NaOH pH 7.5 and 200 mM NaCl, which was connected to the miniDAWN TREOS and Optilab rEX instruments (Wyatt Technologies). Samples were analyzed by multiangle static light scattering, and the absolute molecular weight of each protein was calculated from the light scattering data with ASTRA (Wyatt Technologies).

### Deadenylation assays

Deadenylation assays were carried out as described previously^35,45^ and with minor modifications. Briefly, the deadenylase complex to be tested was mixed with a synthetic RNA substrate (sequences are listed in Supplementary Table 4) carrying a 6-FAM label at the 5′ end (Biomers). Reactions were performed at 37 °C in a buffer containing 20 mM PIPES pH 6.8, 10 mM KCl, 40 mM NaCl, and 2 mM Mg(OAc)_2_. The total reaction volume was 60 µl, the concentration of FAM-labeled RNA 50 nM and the concentration of deadenylase complex 50 nM (or 250 nM in the case of the CCR4a:CAF1 dimer). The reactions were stopped at the indicated time points by adding 180 µl of 2× RNA loading dye (95% (v/v) deionized formamide, 17.5 mM EDTA pH 8, 0.01% (w/v) bromophenol blue). In the case of competition assays with CBM peptides, the indicated final concentrations of the respective MBP-tagged peptide (or MBP as control) were incubated with the protein complex for 15 min before mixing with the RNA substrate, and the deadenylation reactions were stopped 32 min after adding the substrate.

The reaction products were separated on denaturing TBE-urea polyacrylamide gels [20% (w/v) 19:1 acrylamide-bis acrylamide, 7 M urea, 1× TBE (Tris-Borate-EDTA) buffer] at 300 V for 2 h for the 7-mer-A_20_ substrate, and 3.5 h for the longer substrates, followed by analysis using a Typhoon RGB Biomolecular Imager (GE Healthcare).

All deadenylation assay experiments were performed in triplicates. All assays were also extensively validated with proteins and complexes independently purified and reconstituted in different batches.

### Electrophoretic mobility shift assay (EMSA)

Binding reactions contained 100 nM of labeled RNA and 1 µM or 25 μM of the indicated proteins in a total reaction volume of 10 μl of binding buffer (20 mM Tris/HCl 7.5, 10 mM NaCl, 2 mM MgCl_2_, 0.1% BSA, 0.1% Orange G, 3% Ficoll 400). For the competition assay, the reaction mixture contained 100 µM of the NOT1:CAF40 complex and 200 µM of the MBP-tagged Bam CBM (wildtype or mutant as indicated). The proteins were pre-incubated for 30 min before addition of RNA. The RNA-protein complexes were analyzed by electrophoresis on a 10% nondenaturing polyacrylamide gel in TBE buffer, pH 8.3, at 10 V cm^−1^.

### UV crosslinking

Proteins at 1 µM (final concentration) were mixed with 100 nM (final concentration) of 5′ 6-FAM-labeled poly(U)_30_ RNA (Biomers) in a buffer containing 50 mM Tris/HCl pH 7.5, 50 mM ammonium sulfate and 5 mM magnesium chloride in a total reaction volume of 20 µl. The samples to be crosslinked were placed on a precooled rack on ice, and irradiated by a 254 nm UV light source (Stratalinker 2400) at a total energy dose of 2400 mJ/cm^2^. Subsequently, 2× denaturing protein loading buffer was added and the samples resolved on a 4–12% NuPAGE Bis-Tris gel (Thermo Fisher Scientific). The fluorescence of the 6-FAM label on the RNA was detected using a Typhoon RGB Biomolecular Imager (GE Healthcare), followed by Coomassie staining.

### Pulldown assays

Purified MBP or MBP-tagged peptides (30 µg) were incubated with 30 µl amylose resin slurry (New England Biolabs) in binding buffer (50 mM Tris/HCl pH 7.5, 150 mM NaCl) at 4 °C. Following 1 h incubation, the beads were washed three times with binding buffer. Then, 20 µg CCR4-NOT complex was added and the mixture incubated at 4 °C for 1 h. Finally, the beads were washed three times with binding buffer, and the bound proteins eluted using binding buffer supplemented with 25 mM D-(+)-maltose and analyzed by SDS-PAGE.

### Electron microscopy

For EM analysis, CCR4-NOT_FULL_ was applied to EM grids coated with a continuous carbon layer at a concentration of 20 µg/ml and negatively stained using 0.75% (w/v) uranyl formate. The grids were analyzed on a Tecnai G Spirit TEM (Thermo Fisher Scientific) equipped with a TVIPs TemCam F416 4k CMOS camera. Mild crosslinking was performed during sucrose gradient centrifugation using the GraFix protocol^51^ on a gradient of 10–30% (w/v) sucrose. The gradient was fractionated, and the fractions analyzed for protein content via a dot blot using an UltraCruz nitrocellulose membrane (Santa Cruz Biotechnology) and stained by amido black 10B (Sigma Aldrich) [0.1% (w/v) dissolved in 25% (v/v) isopropanol, 10% (v/v) acetic acid].

### Reporting summary

Further information on research design is available in the Nature Research Reporting Summary linked to this article.

## Supplementary information

Supplementary Information

Peer Review

Reporting Summary

Source Data

## Data Availability

A reporting summary for this Article is available as a Supplementary Information file. Source data for Figs. 1b, 2b–h, 3–6 and Supplementary Fig. 1b, d, f, 2b–g, 3, 4b, c, e, 5 and 6 are are provided as a Source Data file. All data is available from the corresponding author upon reasonable request.

## References

[1] Wahle E (1995). Poly(A) tail length control is caused by termination of processive synthesis. J. Biol. Chem..

[2] Subtelny AO, Eichhorn SW, Chen GR, Sive H, Bartel DP (2014). Poly(A)-tail profiling reveals an embryonic switch in translational control. Nature.

[3] Lima SA (2017). Short poly(A) tails are a conserved feature of highly expressed genes. Nat. Struct. Mol. Biol..

[4] Temme C, Simonelig M, Wahle E (2014). Deadenylation of mRNA by the CCR4-NOT complex in Drosophila: molecular and developmental aspects. Front. Genet..

[5] Collart MA (2016). The Ccr4-Not complex is a key regulator of eukaryotic gene expression. Wiley Interdiscip. Rev. RNA.

[6] Yamashita A (2005). Concerted action of poly(A) nucleases and decapping enzyme in mammalian mRNA turnover. Nat. Struct. Mol. Biol..

[7] Yi H (2018). PABP cooperates with the CCR4-NOT complex to promote mRNA deadenylation and block precocious decay. Mol. Cell.

[8] Braun JE, Huntzinger E, Fauser M, Izaurralde E (2011). GW182 proteins directly recruit cytoplasmic deadenylase complexes to miRNA targets. Mol. Cell.

[9] Leppek K (2013). Roquin promotes constitutive mRNA decay via a conserved class of stem-loop recognition motifs. Cell.

[10] Bhandari D, Raisch T, Weichenrieder O, Jonas S, Izaurralde E (2014). Structural basis for the Nanos-mediated recruitment of the CCR4-NOT complex and translational repression. Genes Dev..

[11] Raisch T (2016). Distinct modes of recruitment of the CCR4-NOT complex by Drosophila and vertebrate Nanos. EMBO J..

[12] Van Etten J (2012). Human Pumilio proteins recruit multiple deadenylases to efficiently repress messenger RNAs. J. Biol. Chem..

[13] Sgromo A (2017). A CAF40-binding motif facilitates recruitment of the CCR4-NOT complex to mRNAs targeted by Drosophila Roquin. Nat. Commun..

[14] Chekulaeva M (2011). miRNA repression involves GW182-mediated recruitment of CCR4-NOT through conserved W-containing motifs. Nat. Struct. Mol. Biol..

[15] Fabian MR (2011). miRNA-mediated deadenylation is orchestrated by GW182 through two conserved motifs that interact with CCR4-NOT. Nat. Struct. Mol. Biol..

[16] Sarshad AA (2018). Argonaute-miRNA complexes silence target mrnas in the nucleus of mammalian stem cells. Mol. Cell.

[17] Maillet L, Tu C, Hong YK, Shuster EO, Collart MA (2000). The essential function of Not1 lies within the Ccr4-Not complex. J. Mol. Biol..

[18] Temme C (2010). Subunits of the Drosophila CCR4-NOT complex and their roles in mRNA deadenylation. RNA.

[19] Bawankar P, Loh B, Wohlbold L, Schmidt S, Izaurralde E (2013). NOT10 and C2orf29/NOT11 form a conserved module of the CCR4-NOT complex that docks onto the NOT1 N-terminal domain. RNA Biol..

[20] Sgromo A (2018). Bag-of-marbles directly interacts with the CAF40 subunit of the CCR4-NOT complex to elicit repression of mRNA targets. RNA.

[21] Albert TK (2000). Isolation and characterization of human orthologs of yeast CCR4-NOT complex subunits. Nucleic Acids Res..

[22] Chen J (2001). Purification and characterization of the 1.0 MDa CCR4-NOT complex identifies two novel components of the complex. J. Mol. Biol..

[23] Lau N-C (2009). Human Ccr4-Not complexes contain variable deadenylase subunits. Biochem. J..

[24] Tucker M, Staples RR, Valencia-Sanchez MA, Muhlrad D, Parker R (2002). Ccr4p is the catalytic subunit of a Ccr4p/Pop2p/Notp mRNA deadenylase complex in Saccharomyces cerevisiae. EMBO J..

[25] Nousch M, Techritz N, Hampel D, Millonigg S, Eckmann CR (2013). The Ccr4-Not deadenylase complex constitutes the main poly(A) removal activity in C. elegans. J. Cell Sci..

[26] Temme C, Zaessinger S, Meyer S, Simonelig M, Wahle E (2004). A complex containing the CCR4 and CAF1 proteins is involved in mRNA deadenylation in Drosophila. EMBO J..

[27] Piao X, Zhang X, Wu L, Belasco JG (2010). CCR4-NOT deadenylates mRNA associated with RNA-induced silencing complexes in human cells. Mol. Cell. Biol..

[28] Webster MW (2018). mRNA deadenylation is coupled to translation rates by the differential activities of CCR4-not nucleases. Mol. Cell.

[29] Bai Y (1999). The CCR4 and CAF1 proteins of the CCR4-NOT complex are physically and functionally separated from NOT2, NOT4, and NOT5. Mol. Cell. Biol..

[30] Albert TK (2002). Identification of a ubiquitin-protein ligase subunit within the CCR4-NOT transcription repressor complex. EMBO J..

[31] Mauxion F, Prève B, Séraphin B (2013). C2ORF29/CNOT11 and CNOT10 form a new module of the CCR4-NOT complex. RNA Biol..

[32] Fabian MR (2013). Structural basis for the recruitment of the human CCR4-NOT deadenylase complex by tristetraprolin. Nat. Struct. Mol. Biol..

[33] Chen Y (2014). A DDX6-CNOT1 complex and W-binding pockets in CNOT9 reveal direct links between miRNA target recognition and silencing. Mol. Cell.

[34] Mathys H (2014). Structural and biochemical insights to the role of the CCR4-NOT complex and DDX6 ATPase in microRNA repression. Mol. Cell.

[35] Stowell JAW (2016). Reconstitution of targeted deadenylation by the Ccr4-not complex and the YTH domain protein Mmi1. Cell Rep..

[36] Horiuchi M (2009). Structural basis for the antiproliferative activity of the Tob-hCaf1 complex. J. Biol. Chem..

[37] Wang H (2010). Crystal structure of the human CNOT6L nuclease domain reveals strict poly(A) substrate specificity. EMBO J..

[38] Petit A-P (2012). The structural basis for the interaction between the CAF1 nuclease and the NOT1 scaffold of the human CCR4-NOT deadenylase complex. Nucleic Acids Res..

[39] Boland A (2013). Structure and assembly of the NOT module of the human CCR4-NOT complex. Nat. Struct. Mol. Biol..

[40] Raisch T, Sandmeir F, Weichenrieder O, Valkov E, Izaurralde E (2018). Structural and biochemical analysis of a NOT1 MIF4G-like domain of the CCR4-NOT complex. J. Struct. Biol..

[41] Garces RG, Gillon W, Pai EF (2007). Atomic model of human Rcd-1 reveals an armadillo-like-repeat protein with in vitro nucleic acid binding properties. Protein Sci..

[42] Sari D (2016). The MultiBac Baculovirus/insect cell expression vector system for producing complex protein biologics. Adv. Exp. Med. Biol..

[43] Bieniossek C, Imasaki T, Takagi Y, Berger I (2012). MultiBac: expanding the research toolbox for multiprotein complexes. Trends Biochem. Sci..

[44] Bhaskar V (2013). Structure and RNA-binding properties of the Not1-Not2-Not5 module of the yeast Ccr4-Not complex. Nat. Struct. Mol. Biol..

[45] Webster MW, Stowell JAW, Tang TTL, Passmore LA (2017). Analysis of mRNA deadenylation by multi-protein complexes. Methods.

[46] Niinuma S, Fukaya T, Tomari Y (2016). CCR4 and CAF1 deadenylases have an intrinsic activity to remove the post-poly(A) sequence. RNA.

[47] Pavanello L, Hall B, Airhihen B, Winkler GS (2018). The central region of CNOT1 and CNOT9 stimulates deadenylation by the Ccr4-Not nuclease module. Biochem. J..

[48] Maryati M, Airhihen B, Winkler GS (2015). The enzyme activities of Caf1 and Ccr4 are both required for deadenylation by the human Ccr4-Not nuclease module. Biochem. J..

[49] Jonstrup AT, Andersen KR, Van LB, Brodersen DE (2007). The 1.4-A crystal structure of the S. pombe Pop2p deadenylase subunit unveils the configuration of an active enzyme. Nucleic Acids Res..

[50] Keskeny C (2019). A conserved CAF40-binding motif in metazoan NOT4 mediates association with the CCR4-NOT complex. Genes Dev..

[51] Kastner B (2008). GraFix: sample preparation for single-particle electron cryomicroscopy. Nat. Methods.

[52] Nasertorabi F, Batisse C, Diepholz M, Suck D, Böttcher B (2011). Insights into the structure of the CCR4-NOT complex by electron microscopy. FEBS Lett..

[53] Ukleja M (2016). The architecture of the Schizosaccharomyces pombe CCR4-NOT complex. Nat. Commun..

[54] Lim J (2018). Mixed tailing by TENT4A and TENT4B shields mRNA from rapid deadenylation. Science.

[55] Shirai Y-T, Suzuki T, Morita M, Takahashi A, Yamamoto T (2014). Multifunctional roles of the mammalian CCR4-NOT complex in physiological phenomena. Front. Genet..

[56] Bulbrook D (2018). Tryptophan-mediated interactions between tristetraprolin and the CNOT9 subunit are required for CCR4-NOT deadenylase complex recruitment. J. Mol. Biol..

[57] Donnelly MI (2006). An expression vector tailored for large-scale, high-throughput purification of recombinant proteins. Protein Expr. Purif..

[58] Diebold M-L, Fribourg S, Koch M, Metzger T, Romier C (2011). Deciphering correct strategies for multiprotein complex assembly by co-expression: application to complexes as large as the histone octamer. J. Struct. Biol..

[59] Liu H, Naismith JH (2008). An efficient one-step site-directed deletion, insertion, single and multiple-site plasmid mutagenesis protocol. BMC Biotechnol..

