# Supplementary material for: Reconstitution of recombinant human CCR4-NOT reveals molecular insights into regulated deadenylation
Source: Nat Commun. 2019 Jul 18;10:3173. doi: 10.1038/s41467-019-11094-z (PMC6639331; doi:10.1038/s41467-019-11094-z)
Supplement: Supplementary file 1 — Supplementary Information [file 41467_2019_11094_MOESM1_ESM.pdf]

SUPPLEMENTARY INFORMATION

Reconstitution of recombinant human CCR4-NOT reveals molecular insights into regulated deadenylation

Raisch et al.

Supplementary Table 1. Subunits of the human CCR4-NOT complex.

Subunit	UniProt ID	No. of residues	Molecular mass (kDa)	Synonyms
**NOT1**	A5YKK6-1	2376	266.9	CNOT1
**NOT2**	Q9NZN8-1	540	59.7	CNOT2
**NOT3**	O75175-1	753	81.9	CNOT3
**CAF40**	Q92600-1	299	33.6	CNOT9, RCD-1
**CCR4a**	Q9ULM6-1	557	63.3	CNOT6
**CAF1**	Q9UIV1-1	285	32.7	CNOT7
**NOT10**	Q9H9A5-1	744	82.3	CNOT10
**NOT11**	Q9UKZ1-1	510	55.2	CNOT11, C2ORF29
**Total**		6064	675.6	

Supplementary Table 2. Plasmids used in this study.

Plasmid	Protein	Residues	Tag	Protease site	Restriction sites	Comments
pACEBac-NOT1-CAF40						Acceptor plasmid for recombination with pIDK-NOT2-NOT3
	NOT1	1-2376	His$_{10}$ (N)	TEV	-	
			StrepII$_2$ (C)	-		
	CAF40	1-299	-	-	-	
pIDK-NOT2-NOT3						Donor plasmid for recombination with pACEBac-NOT1-CAF40
	NOT2	1-540	-	-	XhoI/NcoI	
	NOT3	1-753	-	-	KpnI	
pACEBac-NOT1-CAF40-NOT2-NOT3						Bacmid for production of NOT1:NOT2:NOT3:CAF40 complex.<br><br>Created by Cre-Lox recombination of pACEBac-NOT1-CAF40 and pIDK-NOT2-NOT3.<br><br>For reconstitution of CCR4-NOT$_{FULL}$ and CCR4-NOT$_{\Delta10:11}$
	NOT1	1-2376	His$_{10}$ (N)	TEV	-	
			StrepII$_2$ (C)	-		
	CAF40	1-299	-	-	-	
	NOT2	1-540	-	-	XhoI/NcoI	
	NOT3	1-753	-	-	KpnI	
pMCSG19c-CCR4a						Used for production of CCR4a:CAF1 dimer.

	CCR4a	1-557	MBP (N)	TVMV	KpnI/BamHI	For reconstitution of all larger complexes.<br><br>MBP is proteolytically cleaved *in vivo*.
			His$_6$ (N)	TEV		
pET28b-SUMO-CAF1						Used for production of CCR4a:CAF1 dimer.<br><br>For reconstitution of all larger complexes
	CAF1	285	SUMO-His$_6$ (N)	TEV	BamHI/HindIII	
pMCSG19c-CCR4a^{E240A}						Used for production of CCR4a^{E240A}:CAF1 dimer.<br><br>For reconstitution of all larger complexes.<br><br>MBP is proteolytically cleaved *in vivo*.<br><br>Generated from wildtype plasmid using site-directed mutagenesis
	CCR4a	1-557	MBP (N)	TVMV	KpnI/BamHI	
			His$_6$ (N)	TEV		
pET28b-CAF1^{D40A}						Used for production of CCR4a:CAF1^{D40A} dimer.<br><br>For reconstitution of all larger complexes<br><br>Generated from wildtype plasmid using site-directed mutagenesis
	CAF1	285	SUMO-His$_6$ (N)	TEV	BamHI/HindIII	
pnYC-pM-NOT10_25-707						Used for production of NOT10:NOT11 dimer.<br><br>For reconstitution of CCR4-NOT$_{FULL}$
	NOT10	25-707	MBP (N)	HRV-3C	XhoI/BamHI	
pnEA-NOT11_257-498-vH						Used for production of NOT10:NOT11 dimer.<br><br>For reconstitution of CCR4-NOT$_{FULL}$
	NOT11	257-498	His$_6$ (C)	TEV	XhoI/BamHI	
pnYC-pM-NOT1_1093-2376						*Sgromo et al., 2017*<br><br>For reconstitution of CCR4-NOT$_{MINI}$
	NOT1	1093-2376	MBP (N)	HRV-3C	XhoI/BamHI	
pnEA-H-NOT3_607-753-pM-NOT2_344-540-pH-CAF40_19-285						For reconstitution of CCR4-NOT$_{MINI}$

	NOT3	607-753	His_6 (N)	-	NdeI/BamHI	
	NOT2	344-540	MBP (N)	HRV-3C	NdeI/BamHI	
	CAF40	19-285	His_6 (N)	HRV-3C	XhoI/BamHI	
pnYC-pM-NOT1_1093-1607						For reconstitution of CCR4-NOT_{CORE}
	NOT1	1093-1607	MBP (N)	HRV-3C	XhoI/BamHI	
pnEA-CAF40_19-285						For reconstitution of CCR4-NOT_{CORE}
	CAF40	19-285	-	-	XhoI/BamHI	
	NOT1	1-682	-	-	-	
pnYC-pM-NOT1_1351-1588						For production of CAF40 module
	NOT1	1351-1588	MBP (N)	HRV-3C	XhoI/BamHI	
pnEA-pH-CAF40_19-285						*Chen et al., 2014*<br>For production of CAF40 module
	CAF40	19-285	His_6 (N)	HRV-3C	XhoI/BamHI	
pnYC-pM-NOT1_1833-2361						*Bhandari et al., 2014*<br>For production of NOT module
	NOT1	1833-2361	MBP (N)	HRV-3C	XhoI/BamHI	
pnEA-pH-NOT3_607-748-pM-NOT2_350-540						*Bhandari et al., 2014*<br>For production of NOT module
	NOT3	607-748	His_6 (N)	HRV-3C	XhoI/BamHI	

	NOT2	350-540	MBP (N)	HRV-3C	XhoI/BamHI	
pnYC-vM-DmBam-CBM						*Sgromo et al., 2018*
	Bam	13-36	MBP (N)	TEV	XhoI/AvrII	
pnYC-vM-DmBam-CBM-Mut						Contains L17E and M24E mutations<br>Generated from wildtype plasmid using site-directed mutagenesis
	Bam	13-36	MBP (N)	TEV	XhoI/AvrII	
pnYC-pM-Roquin1-C						*Sgromo et al., 2018*
	Roquin1	501-1133	MBP (N)	HRV-3C	AflII/AvrII	
pnEA-pM-NOT4-CBM						*Keskeny et al., 2019*
	NOT4	400-427	MBP (N)	HRV-3C	XhoI/NheI	
pnEA-pM-CAF40_18-293						For UV crosslinking
	CAF40	18-293	MBP (N)	HRV-3C	XhoI/BamHI	

Supplementary Table 3. Complexes investigated in this study.

**CCR4-NOT$_{FULL}$**				
**Subunit**	**Expression host**	**Residues**	**Mol. weight**	**Comment**
**NOT1**	Sf21 insect cells	2408	270.7 kDa	Full-length, N-terminal His_{10} tag, C-terminal $StrepII_2$ tag
**NOT2**	Sf21 insect cells	540	59.7 kDa	Full-length
**NOT3**	Sf21 insect cells	753	81.9 kDa	Full-length
**CAF40**	Sf21 insect cells	299	33.6 kDa	Full-length
**CCR4a**	*E. coli* BL21(DE3) Star	558	63.4 kDa	Full-length, N-terminal His_6 tag (proteolytically removed)
**CAF1**	*E. coli* BL21(DE3) Star	286	32.8 kDa	Full-length, N-terminal His_6-SUMO tag (proteolytically removed)
**NOT10**	*E. coli* BL21(DE3) Star	689	76.4 kDa	Residues 25-707, N-terminal MBP tag (proteolytically removed)
**NOT11**	*E. coli* BL21(DE3) Star	253	28.8 kDa	Residues 257-498, C-terminal His_6 tag (proteolytically removed)
**Total**		5786	647.3 kDa	
**CCR4-NOT$_{Δ10:11}$**				
**Subunit**	**Expression host**	**Residues**	**Mol. weight**	**Comment**
**NOT1**	Sf21 insect cells	2408	270.7 kDa	Full-length, N-terminal His_{10} tag, C-terminal $StrepII_2$ tag
**NOT2**	Sf21 insect cells	540	59.7 kDa	Full-length

**NOT3**	Sf21 insect cells	753	81.9 kDa	Full-length
**CAF40**	Sf21 insect cells	299	33.6 kDa	Full-length
**CCR4a**	*E. coli* BL21(DE3) Star	558	63.4 kDa	Full-length, N-terminal His_6 tag (proteolytically removed)
**CAF1**	*E. coli* BL21(DE3) Star	286	32.8 kDa	Full-length, N-terminal His_6-SUMO tag (proteolytically removed)
**Total**		4844	542.1 kDa	
**CCR4-NOT$_{MINI}$**				
**Subunit**	**Expression host**	**Residues**	**Mol. weight**	**Comment**
**NOT1**	*E. coli* BL21(DE3) Star	1292	146.9 kDa	Residues 1093-2376, N-terminal His_6 tag (proteolytically removed)
**NOT2**	*E. coli* BL21(DE3) Star	201	23.3 kDa	Residues 344-540, N-terminal MBP tag (proteolytically removed)
**NOT3**	*E. coli* BL21(DE3) Star	166	20.2 kDa	Residues 607-753, N-terminal His_6 tag
**CAF40**	*E. coli* BL21(DE3) Star	273	31.0 kDa	Residues 19-285, N-terminal His_6 tag (proteolytically removed)
**CCR4a**	*E. coli* BL21(DE3) Star	558	63.4 kDa	Full-length, N-terminal His_6 tag (proteolytically removed)
**CAF1**	*E. coli* BL21(DE3) Star	286	32.8 kDa	Full-length, N-terminal His_6-SUMO tag (proteolytically removed)
**Total**		2776	317.6 kDa	

CCR4-NOT$_{CORE}$				
**Subunit**	**Expression host**	**Residues**	**Mol. weight**	**Comment**
**NOT1**	*E. coli* BL21(DE3) Star	521	59.2 kDa	Residues 1093-1607, N-terminal MBP tag (proteolytically removed)
**CCR4a**	*E. coli* BL21(DE3) Star	558	63.4 kDa	Full-length, N-terminal His_6 tag (proteolytically removed)
**CAF1**	*E. coli* BL21(DE3) Star	286	32.8 kDa	Full-length, N-terminal His_6-SUMO tag (proteolytically removed)
**CAF40**	*E. coli* BL21(DE3) Star	270	30.7 kDa	Residues 19-285
**Total**		1635	186.1 kDa	
**NOT10:NOT11 heterodimer**				
**Subunit**	**Expression host**	**Residues**	**Mol. weight**	**Comment**
**NOT10**	*E. coli* BL21(DE3) Star	689	76.4 kDa	Residues 25-707, N-terminal MBP tag (proteolytically removed)
**NOT11**	*E. coli* BL21(DE3) Star	253	28.8 kDa	Residues 257-498, C-terminal His_6 tag (proteolytically removed)
**Total**		942	105.2 kDa	
**CCR4a:CAF1 exonuclease heterodimer**				
**Subunit**	**Expression host**	**Residues**	**Mol. weight**	**Comment**
**CCR4a**	*E. coli* BL21(DE3) Star	558	63.4 kDa	Full-length, N-terminal His_6 tag (proteolytically removed)
**CAF1**	*E. coli* BL21(DE3) Star	286	32.8 kDa	Full-length, N-terminal His_6-SUMO tag (proteolytically removed)

**Total**		844	96.2 kDa	
**CAF40 module**				
**Subunit**	**Expression host**	**Residues**	**Mol. weight**	**Comment**
**NOT1**	*E. coli* BL21(DE3) Star	244	27.6 kDa	Residues 1351-1588, N-terminal MBP tag (proteolytically removed)
**CAF40**	*E. coli* BL21(DE3) Star	273	31.0 kDa	Residues 19-285, N-terminal His_6 tag (proteolytically removed)
**Total**		517	58.6 kDa	
**NOT module**				
**Subunit**	**Expression host**	**Residues**	**Mol. weight**	**Comment**
**NOT1**	*E. coli* BL21(DE3) Star	535	61.5 kDa	Residues 1833-2361, N-terminal MBP tag (proteolytically removed)
**NOT2**	*E. coli* BL21(DE3) Star	197	22.9 kDa	Residues 350-540, N-terminal MBP tag (proteolytically removed)
**NOT3**	*E. coli* BL21(DE3) Star	148	18.2 kDa	Residues 607-748, N-terminal His_6 tag (proteolytically removed)
**Total**		880	102.6 kDa	

Supplementary Table 4. List of RNA substrates.

Name	Sequence	Comments
**7-mer-A_{20}**	UCUAAAUAAAAAAAAAAAAAAAAAAAA	AAUAAA - sequence used by Wang et al., 2010
**13+7-mer-A_{20}**	CACAUCCAACUUCUCUAAAUAAA AAAAAAAAAAAAAAAAA	Underlined sequence is the 7-mer above. 13 bases predicted to be unstructured are 5’ terminal to the 7-mer.
**13-SL-7mer-A_{20}**	CA**AGAGA**AUCC**UCUCU**AAAUAAA AAAAAAAAAAAAAAAAA	Residues highlighted in bold base-pair to form a stem-loop.
**20-mer-A_{20}**	CACAUCCAACUUCACUUAUCAAA AAAAAAAAAAAAAAAAA	Underlined sequence is altered compared to 7-mer-A_{20} with preceding 13 bases identical to 13-US-7-mer-A_{20}
**4G-20-mer-A_{20}**	CA***G***AUCCAA***G***UU***A***A***G***UUAU***G***AAA AAAAAAAAAAAAAAAAA	Derived from 20-mer-A_{20}; the G nucleotide substitutions are highlighted in bold italic

Supplementary Table 5. List of DNA oligonucleotide primers used in this study.

Name	Sequence (5′—3′)	Description
EV048	ATTATCTCCATGATCTATTAATATTCCGGGCAGCCGGATCTTCTA GGC	SV40/polh cassette assembly
EV049	GCCTAGAAGATCCGGCTGCCCGGAATATTAATAGATCATGGAGA TAAT	SV40/polh cassette assembly
EV053	TGAACCTGAAACATAAAATGAATGC	SV40/polh cassette assembly
EV056	GCGCCCGATGGTGGGACG	SV40/polh cassette assembly
EV051	CGTCCCACCATCGGGCGCATGCACAGCCTGGCGACGGCTG	NOT9 forward
EV052	TCACTGAGGGGGCAGGGG	NOT9 reverse
EV155	GATCGTCGACATGGCCCACCACCACCACCACCACCACCACCACC ACGAGAACCTGTACTTCCAGGGCATGAATCTTGACTCGCTCTCG	NOT1 forward
EV156	GATCGCGGCCGCGCATTCATTTTATGTTTCAGGTTCATCACTTCT CGAACTGGGGGTGGCTCCAGCCGCCGCTGCCGCCACTGGCACC TGTCCCTTCC	NOT1 reverse
EV157	GATCTCTAGATGAACCTGAAACATAAAATGAATGCAATT	NOT9 cassette forward
EV158	GATCAAGCTTTCACTGAGGGGGCAGGGGGATAC	NOT9 cassette reverse
EV149	GATCGGTACCATGGCGGACAAGCGCAAACTCCAAG	NOT3 forward
EV150	GATCGGTACCTCACTGGAGGTCCCGGTCCTCC	NOT3 reverse
EV147	GATCCTCGAGATGGTGAGGACTGATGGACATACATTATCT	NOT2 forward
EV148	GATCCCATGGTTAGAAGGCTTGCTGAGCAGGG	NOT2 reverse
EV075	TCTCATGCTGGAGTTCTTCGC	NOT2 cassette forward
EV076	GATCATCGATGGGTGGGGAAAAGGAAGAAAC	NOT2 cassette reverse
EV412	GATCGGATCCGAAAACCTTTACTTCCAGGGCATGCCAGCGGCAA CTGTAGATCATA	CAF1 forward
EV413	GATCAAGCTTTCATGACTGCTTGTTGGCTTCCTC	CAF1 reverse
EV408	GATCGGTACCGAAAACCTTTACTTCCAGGGCATGCCCAAAGAAA AATACGAGCC	CCR4a forward
EV409	GATCGGATCCCTACCTCCTGCCAGGAAGGTGG	CCR4a reverse
TR367	TACGCCCTCGAGGATCAAGAGAAGGAGTTATCCAC	NOT10 forward
TR372	CGTAATGGATCCTTACTGATTCCTTTTGATGATCTGTAAG	NOT10 reverse
TR361	TACGCCCTCGAGGACAGCTCAGTTGCCTCTCAG	NOT11 forward
TR711	CGTAATGGATCCATCCAATGTCTTCAACAACCGG	NOT11 reverse

Supplementary Figure 1

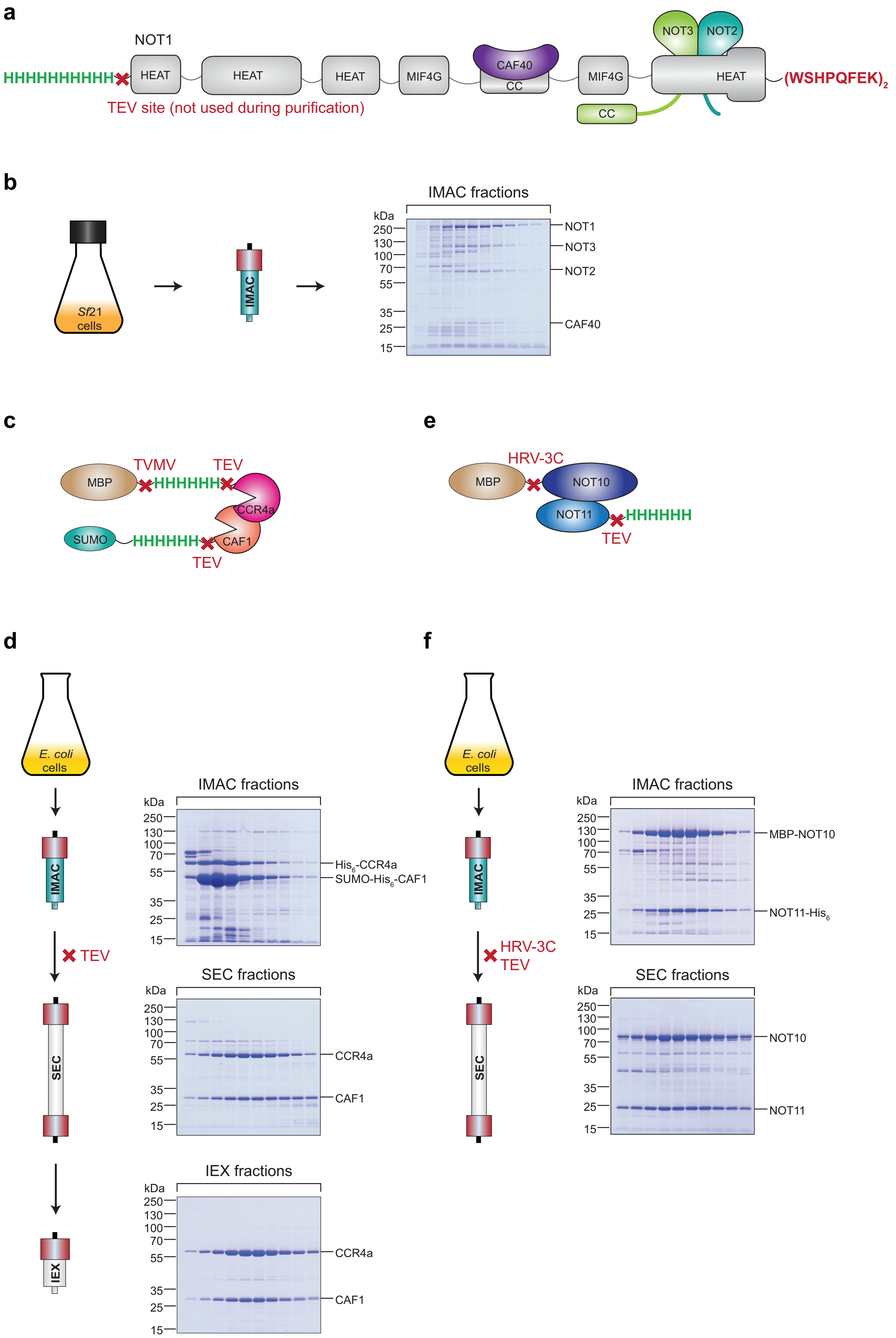

Supplementary Figure 1. Production of subcomplexes used for CCR4-NOT$_{FULL}$ reconstitution.

(a) Schematic representation of the NOT1:NOT2:NOT3:CAF40 subcomplex in the same style as in Fig. 1c.

(b) Purification of the NOT1:NOT2:NOT3:CAF40 subcomplex. The four proteins were co-produced in *Sf*21 cells and isolated from the crude lysate by immobilized metal affinity chromatography (IMAC) via an N-terminal His_{10} tag on NOT1. Fractions containing the complex in sufficient purity were used for reconstitution with the other subcomplexes.

(c) Schematic representation of the CCR4a:CAF1 exonuclease heterodimer.

(d) Purification of the CCR4a:CAF1 heterodimer. Both proteins were co-produced in *E. coli*. The complex was isolated from the crude lysate by IMAC via His_6 tags on both proteins. Both tags were proteolytically removed and the complex separated from the tags by size exclusion chromatography (SEC). High-resolution anion exchange chromatography was used as the final purification step. Representative gels of all three purification steps illustrate the monitoring of the purification.

(e) Schematic representation of the NOT10:NOT11 heterodimer comprising NOT10 (residues 25-707) and NOT11 (residues 257-498).

(f) Purification of the NOT10:NOT11 heterodimer. Both proteins were co-produced in *E. coli*. The complex was isolated from the crude lysate by IMAC via a His_6 tag on NOT11. The His_6 tag from NOT11 and MBP tag from NOT10 were proteolytically removed and the heterodimer purified further by SEC. Representative gels of both purification steps illustrate the monitoring of the purification.

Source data for panels (b,d,f) are provided as a Source Data file.

Supplementary Figure 2

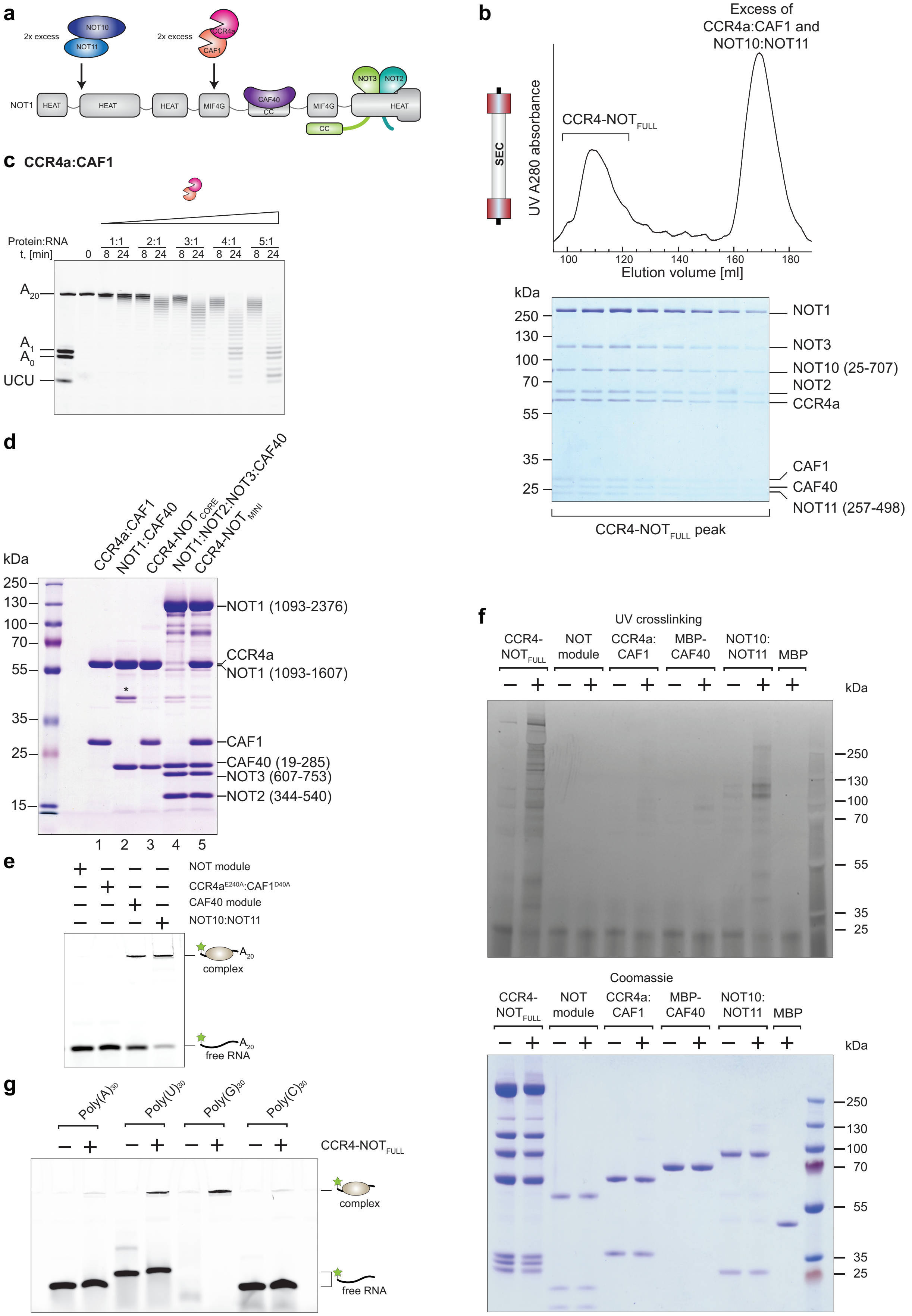

Supplementary Figure 2. Reconstitution of recombinant CCR4-NOT complexes.

(a) Schematic representation of reconstitution of the CCR4-NOT_{FULL} complex. The purified NOT1:NOT2:NOT3:CAF40 subcomplex (Supplementary Fig. 1a,b) was incubated with a two-fold molar excess of purified CCR4a:CAF1 (Supplementary Fig. 1c,d) and NOT10:NOT11 (Supplementary Fig. 1e,f) heterodimers.

(b) Purification of the reconstituted CCR4-NOT_{FULL} complex. After reconstitution (a), the assembled complex was separated from the excess of NOT10:NOT11 and CCR4a:CAF1 heterodimers by SEC. The purity and stoichiometry of the reconstituted CCR4-NOT_{FULL} was verified by SDS-PAGE.

(c) Titration experiment with 50 nM of the 7-mer-A_{20} RNA and the CCR4a:CAF1 dimer in the indicated molar ratios. Increasing the protein:RNA ratio leads to a progressive increase in deadenylation rate. For each ratio, two time points at 8 and 24 minutes, respectively, were analyzed.

(d) SDS-PAGE showing the purified recombinant CCR4a:CAF1 and NOT1:CAF40 complexes used for reconstitution of the CCR4-NOT_{CORE} complex, as well as the CCR4-NOT_{MINI} complex and the corresponding NOT1:NOT2:NOT3:CAF40 subcomplex used for reconstitution. Residual MBP co-purified with the NOT1:CAF40 complex is marked with an asterisk.

(e) Electrophoretic mobility shift assay (EMSA) with 100 nM of the 7-mer-A_{20} RNA and 25 μM CCR4-NOT subcomplexes. The CAF40 module and the NOT10:NOT11 heterodimer bind the RNA as visible from the shift of the RNA band to apparent higher molecular weight in the respective lane. The upshifted protein-RNA complex did not enter the gel.

(f) UV crosslinking experiment with 1 μM CCR4-NOT_{FULL} (CCR4a^{E240A}:CAF1^{D40A}) and subcomplexes, and 100 nM 6-FAM-labelled poly$(U)_{30}$ RNA analysed by denaturing SDS-PAGE. The lower panel shows the Coomassie-stained protein bands, and the upper panel the RNA substrate fluorescence of the same gel.

(g) EMSA with 100 nM of the four homopolymeric 30-mer RNAs and 1 μM CCR4-NOT_{FULL} (CCR4a^{E240A}:CAF1^{D40A}). The complex interacts with poly$(U)_{30}$ and poly$(G)_{30}$ substrates, and only weakly with poly$(A)_{30}$ and poly$(C)_{30}$.

Source data for panels (b-g) are provided as a Source Data file.

Supplementary Figure 3

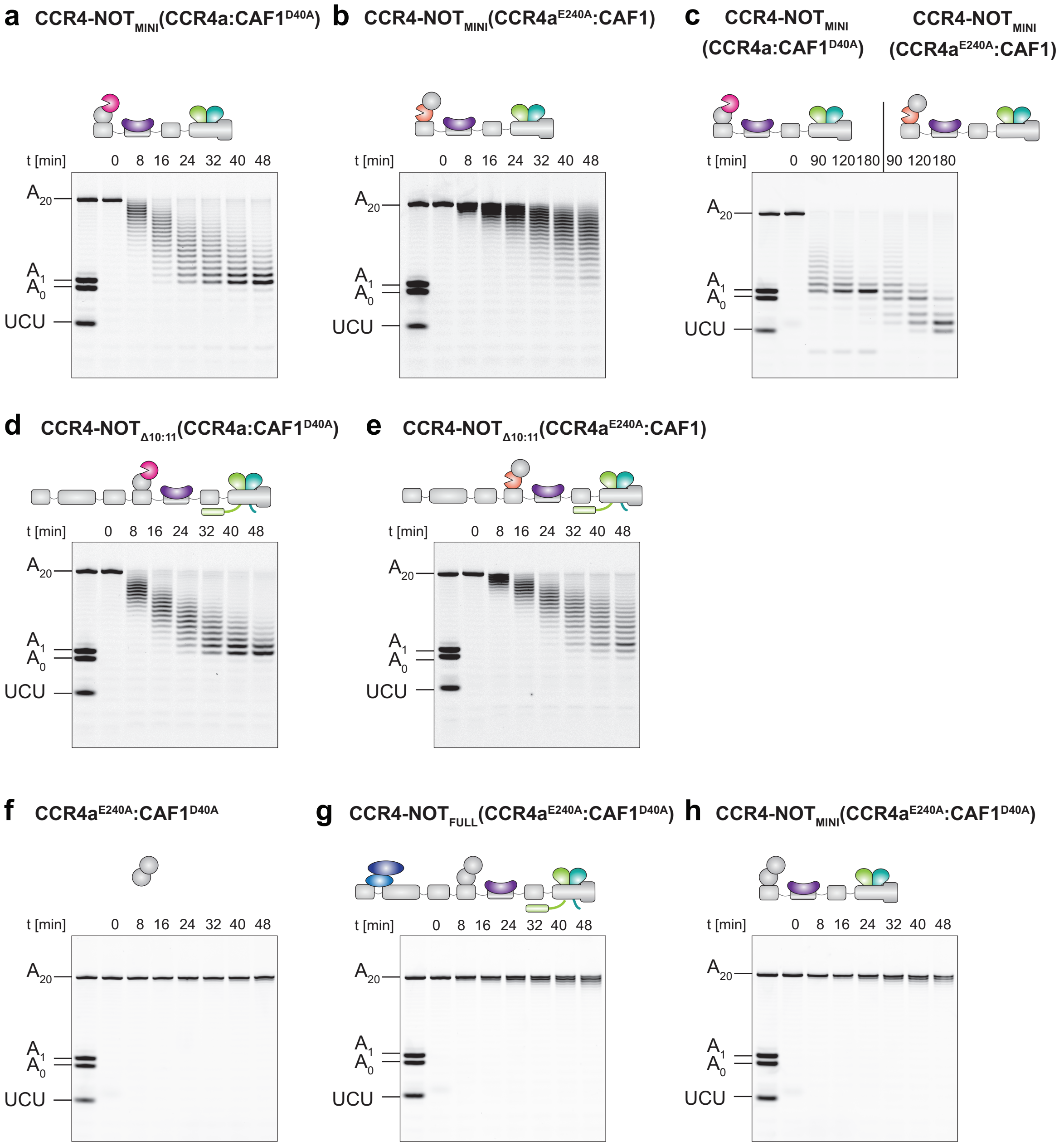

Supplementary Figure 3. Comparison of deadenylation activities of CAF1 and CCR4a.

(a-b) Deadenylation assays with equimolar concentrations (50 nM) of the 7-mer-A_{20} RNA substrate and the CCR4-NOT_{MINI} complex containing inactivating mutations in either CAF1 (a; D40A mutation) or CCR4a (b; E240A mutation), respectively. The $CAF1^{D40A}$ mutation mildly decreases deadenylation rate and enhances selectivity for adenosine compared to wildtype (Fig. 2f), while the $CCR4a^{E240A}$ mutation drastically reduces the deadenylation rate.

(c) Deadenylation assay with equimolar concentrations (50 nM) of the 7-mer-A_{20} RNA substrate and the CCR4-NOT_{MINI} complex containing inactivating mutations in either CAF1 (D40A mutation) or CCR4a (E240A mutation), respectively, incubated longer than in the experiments in (a) and (b). The complex containing the $CAF1^{D40A}$ mutation efficiently stops at A_1, whereas the complex with the $CCR4a^{E240A}$ mutation is able to degrade the substrate down to the UCU trinucleotide.

(d-e) Deadenylation assays with equimolar concentrations (50 nM) of 7-mer-A_{20} RNA and CCR4-$NOT_{\Delta 10:11}$ complexes which contain the same inactivating mutations in CAF1 (d) and CCR4a (e) as above. In the context of this complex, the $CAF1^{D40A}$ mutation mildly decreases deadenylation rate and leads to enhanced selectivity for adenosine (Fig. 2e), while the $CCR4a^{E240A}$ mutation strongly reduces deadenylation activity.

(f-h) Deadenylation assays with 50 nM 7-mer-A_{20} RNA and either 250 nM of the $CCR4a^{E240A}$:$CAF1^{D40A}$ double mutant nuclease dimer (f), or 50 nM of either CCR4-NOT_{FULL} (g) or CCR4-NOT_{MINI} (h) complexes reconstituted with the same double inactive nuclease dimer. In all three cases the complexes retain almost no catalytic activity.

Source data are provided as a Source Data file.

Supplementary Figure 4

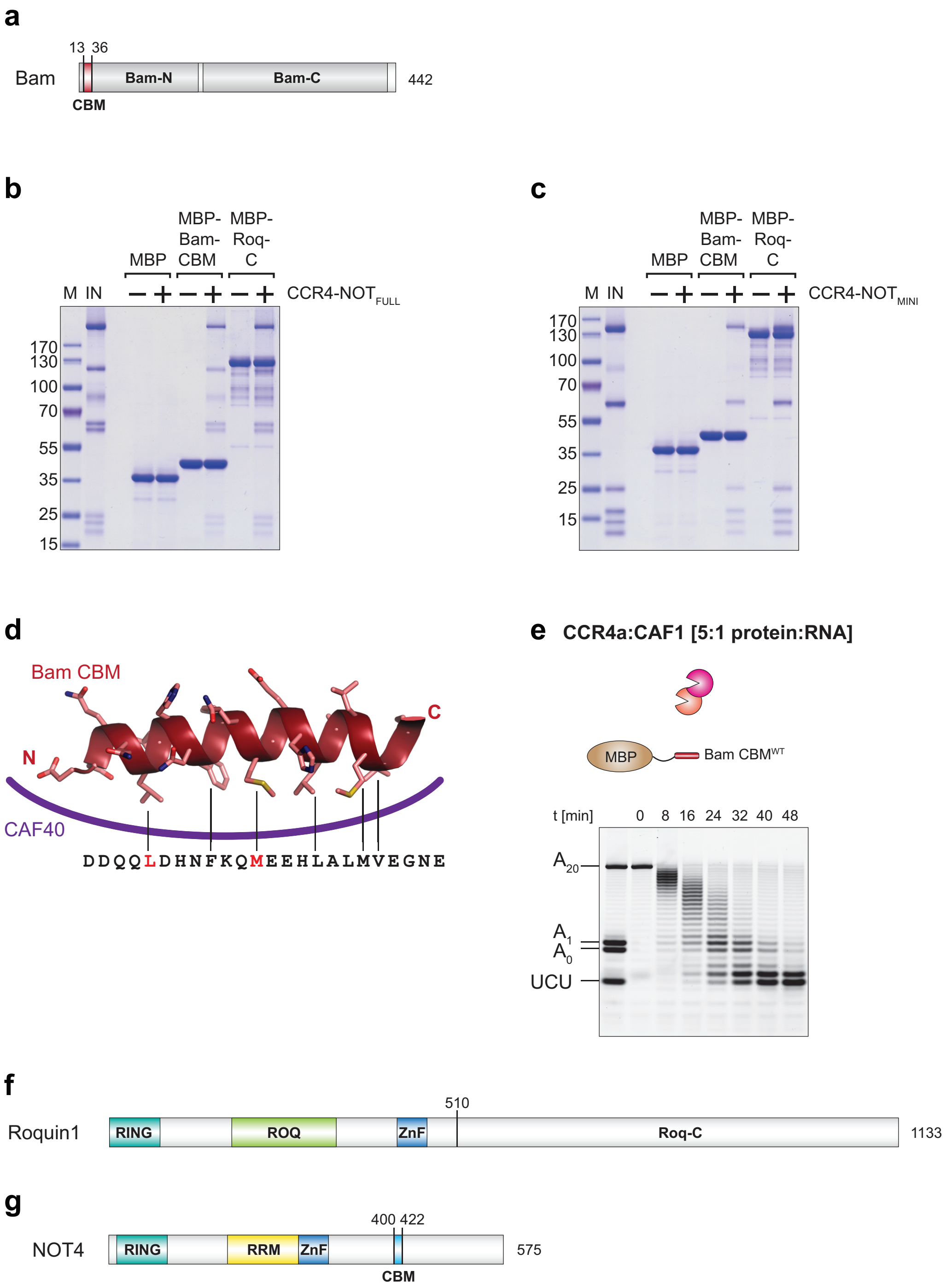

Supplementary Figure 4. CCR4-NOT recruitment factors inhibit deadenylation.

(a) Schematic representation of *Drosophila* Bag-of-marbles (Bam). The protein consists of N- and C-terminal portions (Bam-N and Bam-C, respectively) and contains an N-terminal CAF40-binding motif (CBM)[20].

(b) MBP pulldown assay with either MBP or MBP-tagged Bam CBM or Roquin C-terminal fragment, respectively, and the CCR4-NOT_{FULL} complex. Both peptides specifically interact with CCR4-NOT_{FULL}.

(c) MBP pulldown assay with either MBP or MBP-tagged Bam CBM or Roquin C-terminal fragment, respectively, and the CCR4-NOT_{MINI} complex. Both peptides specifically interact with CCR4-NOT_{MINI}.

(d) Structure of the Bam CBM peptide bound to CAF40. The CBM folds into an amphipathic helix which binds to CAF40, here depicted by a purple line. The sequence of the CBM is shown below, with the two residues mutated in this study highlighted in red.

(e) Time course assay with 50 nM 7-mer-A_{20} RNA, 250 nM CCR4a:CAF1 heterodimer and 12.5 µM MBP-tagged Bam CBM^{WT}. The five-fold higher absolute concentration of the Bam peptide compared to the other experiments was chosen to achieve an equivalent 50-fold molar excess of the peptide over the nuclease dimer or nuclease-containing complex in all cases. The addition of the peptide does not affect the deadenylation activity of the exonuclease heterodimer. The equivalent experiment in the absence of the inhibitory Bam CBM^{WT} peptide is shown in Fig. 2c.

(f) Schematic representation of human Roquin1 consisting of a RING-type E3 ubiquitin ligase domain, a ROQ RNA-binding domain and a zinc finger domain (ZnF) in the N-terminal half of the protein, and an unstructured C-terminal tail which is known to interact with the NOT module and CAF40, even though no Bam-analogous CBM has been identified so far[13].

(g) Schematic representation of human NOT4 containing a RING-type E3 ubiquitin ligase domain, an RNA recognition motif domain (RRM) and a zinc finger domain (ZnF) in the N-terminal region of the protein. A CBM is present within the unstructured C-terminal region of the protein[50].

Source data for panels (b,c,e) are provided as a Source Data file.

Supplementary Figure 5

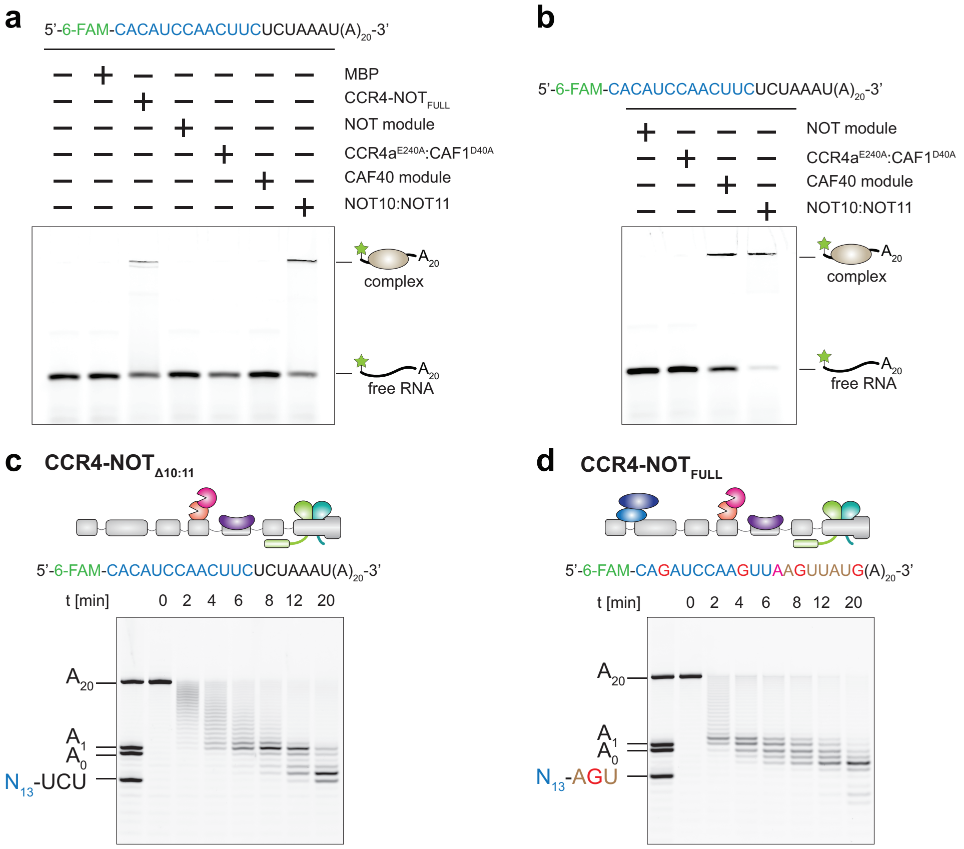

Supplementary Figure 5. CCR4-NOT efficiently binds and deadenylates RNAs with longer bodies.

(a) Electrophoretic mobility shift assay (EMSA) with 100 nM 13+7-mer-A_{20} RNA and 1 µM CCR4-NOT_{FULL} ($CCR4a^{E240A}$:$CAF1^{D40A}$) and subcomplexes. The CCR4-NOT_{FULL} ($CCR4a^{E240A}$:$CAF1^{D40A}$) complex and the NOT10:NOT11 heterodimer both bind the RNA as visible from the shift of the RNA band to apparent higher molecular weight in the respective lanes. The upshifted protein-RNA complexes did not enter the gel.

(b) Electrophoretic mobility shift assay (EMSA) with 100 nM 13+7-mer-A_{20} RNA and 25 µM CCR4-NOT subcomplexes. The CAF40 module and the NOT10:NOT11 heterodimer bind the RNA as visible from the shift of the RNA band to apparent higher molecular weight in the respective lane. The upshifted protein-RNA complex did not enter the gel.

(c) Deadenylation assay with the CCR4-$NOT_{\Delta10:11}$ complex and the 13+7-mer-A_{20} substrate (both 50 nM) with high resolution of the early time points.

(d) Deadenylation assay with an equimolar ratio (50 nM) of the CCR4-NOT_{FULL} complex and an RNA with a 20-mer body containing several guanosine nucleotides, with high resolution of the early time points.

Source data are provided as a Source Data file.

Supplementary Figure 6

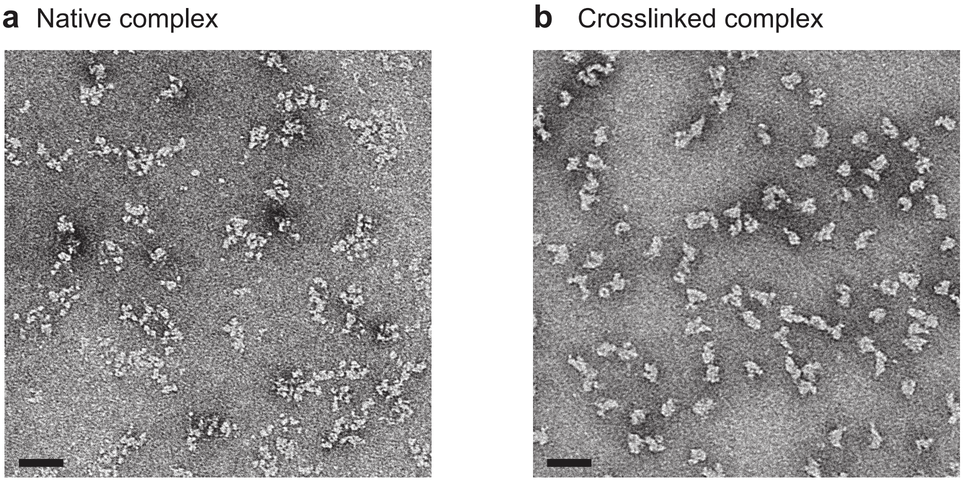

Supplementary Figure 6. Analysis of CCR4-NOT$_{FULL}$ by negative stain electron microscopy.

(a-b) Negative stain electron micrographs of reconstituted native (a) and crosslinked (b) CCR4-NOT$_{FULL}$, imaged on a Tecnai G Spirit electron microscope (Thermo Fisher Scientific) at a nominal magnification of 52,000. Scale bars: 50 nm. Crosslinking by glutaraldehyde during a sucrose gradient stabilizes the complex, but particles are still too heterogeneous for further characterization. Source data are provided as a Source Data file.

Supplementary Figure 7

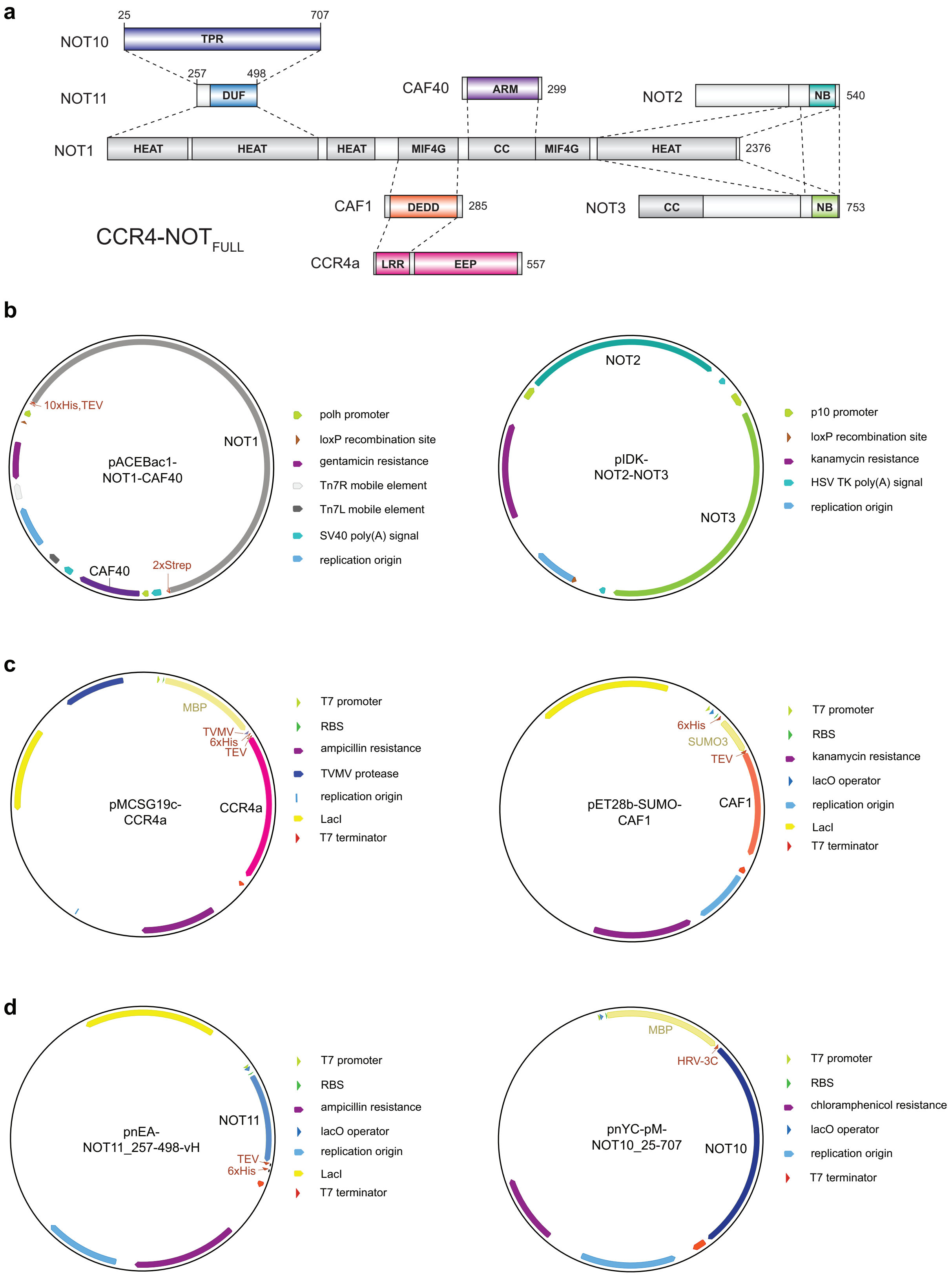

Supplementary Figure 7. Plasmids used for the production of the CCR4-NOT$_{FULL}$ complex.

(a) Schematic representation of the CCR4-NOT$_{FULL}$ complex in the same style as shown in Fig. 1a. All proteins were expressed as full-length constructs, apart from NOT10 (residues 25-707) and NOT11 (residues 257-498).

(b-d) Schematic representations of plasmids used for reconstitution of the CCR4-NOT$_{FULL}$ complex. pACEBac1-NOT1-CAF40 and pIDK-NOT2-NOT3 (b) were assembled using Cre-based recombination and used for generating a recombinant baculovirus for production of the NOT1:NOT2:NOT3:CAF40 subcomplex in *Sf*21 cells. *E. coli* were co-transformed with pMCS19c-CCR4a and pET28b-SUMO-CAF1 (c) for production of the CCR4a:CAF1 heterodimer, and pnEA-NOT11_257-498-vH and pnYC-pM-NOT10_25-707 (d) were co-transformed in *E. coli* for production of the NOT10:NOT11 heterodimer.

Supplementary Figure 8

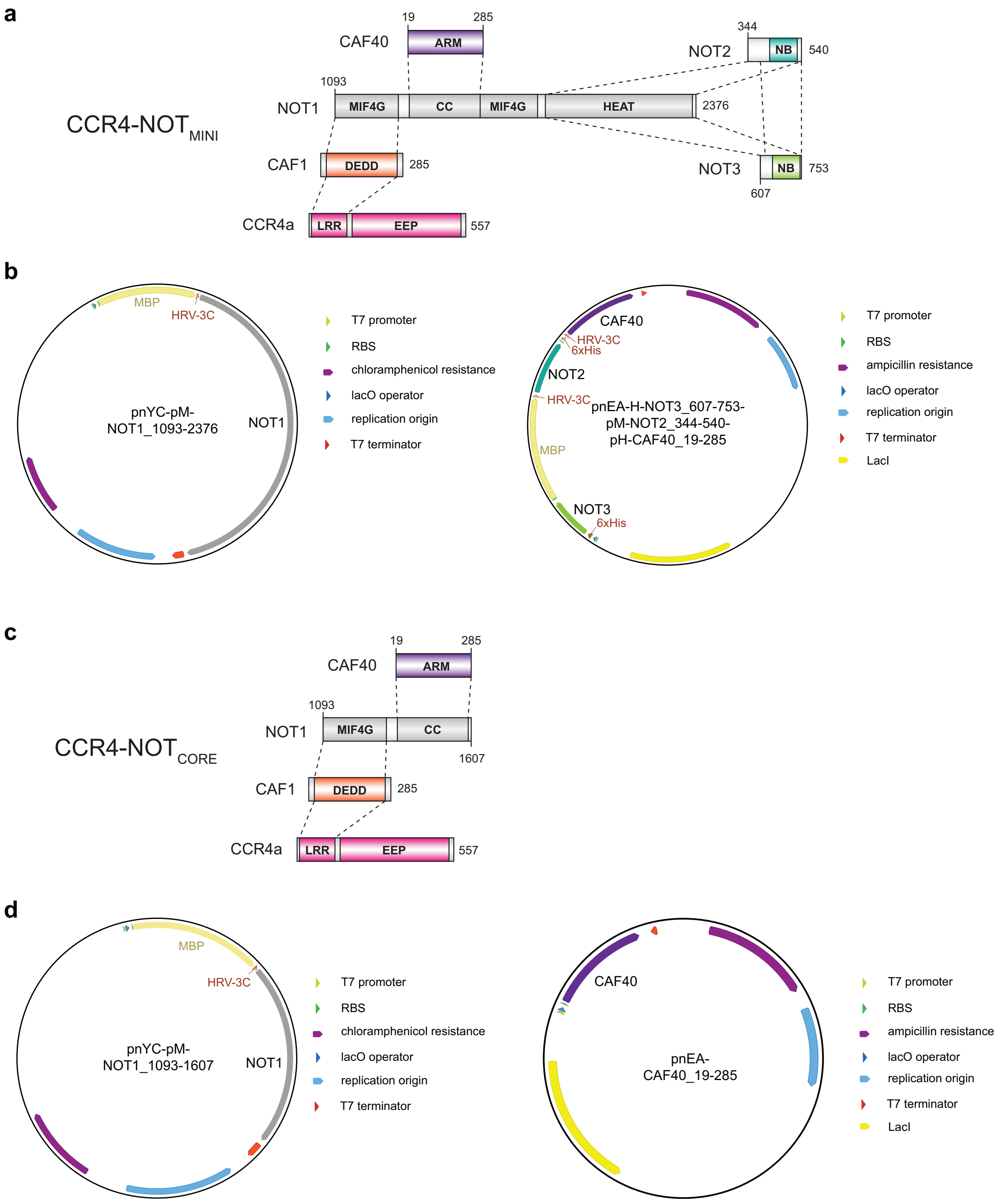

Supplementary Figure 8. Plasmids used for the production of CCR4-NOT$_{MINI}$ and CCR4-NOT$_{CORE}$.

(a) Schematic representation of the CCR4-NOT$_{MINI}$ complex in the same style as shown in Fig. 1a. Construct boundaries of the subunits are indicated.

(b) Schematic representations of plasmids used for reconstitution of the CCR4-NOT$_{MINI}$ complex. *E. coli* were co-transformed with pnYC-pM-NOT1_1093-2376 and pnEA-H-NOT3_607-753-pM-NOT2_344-540-pH-CAF40_19-285 for production of the NOT1:NOT2:NOT3:CAF40 subcomplex.

(c) Schematic representation of the CCR4-NOT$_{CORE}$ complex in the same style as shown in Fig. 1a. The complex contained full-length CCR4a and CAF1, and NOT1 (residues 1093-1607) and CAF40 (residues 19-285)

(d) Schematic representations of plasmids used for reconstitution of the CCR4-NOT$_{CORE}$ complex. *E. coli* were co-transformed with pnYC-pM-NOT1_1093-1607 and pnEA-CAF40_19-285 for production of the NOT1:CAF40 subcomplex.
