# Supplementary material for: Reconstitution of recombinant human CCR4-NOT reveals molecular insights into regulated deadenylation
Source: Nat Commun. 2019 Jul 18;10:3173. doi: 10.1038/s41467-019-11094-z (PMC6639331; doi:10.1038/s41467-019-11094-z)
Supplement: Supplementary file 2 — Peer Review [file 41467_2019_11094_MOESM2_ESM.pdf]

Reviewers' comments:

Reviewer #1 (Remarks to the Author):

Raisch et al. present a highly interesting and very detailed study of the function of the human CCR4-NOT complex based mainly on in vitro degradation of RNA. Using carefully assembled complexes and sub-complexes, the authors are able to dissect the separate catalytic and RNA-binding functions of the complex and start to answer some of the many questions regarding this important deadenylation complex.

The purification and reconstitution of the human CCR4-NOT complex as presented in this paper constitutes a monumental and very impressive accomplishment for which the authors should be acknowledged. Several of the proteins involved contain unstructured regions and handling these as well as reconstitution of such a large, eukaryotic protein complex is in no way trivial. It provides the authors with a unique tool to study the activity and RNA-binding capabilities of CCR4-NOT in vitro, as well as work with subcomplexes and mutants that have never before been studied.

The manuscript is overall very well written and the figures are generally clear and beautifully laid out. That being said, my main concern with the manuscript is that it relies almost exclusively on a single technique, namely in vitro RNA degradation assays using defined, 5' fluorescently labelled RNA substrates. While this technique can give useful insights into specific preferences for RNA sequence and structure, it also involves a risk of over-interpretation of subtle changes in degradation patterns that may or may not have biological relevance.

Major issues

1. The first thing that strikes me is that the model RNA used only contains a "body" (i.e. non-poly-A part) of 7 nucleotides, which is very short given the relatively large size of the CCR4-NOT complex. I think it is possible that the complex might recognise or interact with non-poly-A sequence outside of this and would like the authors to confirm experimentally that other model RNAs with longer bodies behave in a similar fashion to the one used here. For example, when (on page 8) the authors conclude that the NOT10:NOT11 module but not the NOT module interacts with the 7-mer-A20 RNA, would that be true also for a longer model RNA?
2. When working with RNA and purified nucleases there is always the risk of co-purifying small amounts of endogenous nucleases from the expression host, whether E. coli or insect cells. I would therefore like to see a control in Fig. 2b where both active sites of CCR4 and Caf1 have been inactivated showing that in that case there is no RNA degradation. This can easily be achieved by combination of the protein samples shown in Fig. 3.
3. Also in Fig. 2, the authors conclude that the activity of the isolated CCR4:Caf1 heterodimer is not very high and that a 5:1 ratio of protein to RNA needs to be used. This might seem extreme for an enzyme to have to be in excess of its substrate, but is indeed what has been observed in vitro for other deadenylases. However, when looking at the mutants in Fig. 3a/b it needs to be clearer what ratio of protein:RNA is used (I couldn't find this in the legend). I assume this is also 5:1, otherwise the experiments are not consistent with Fig. 2.
4. As mentioned above, relying too much on a single technique can be misleading and I think that the authors in the later figures, especially Fig. 4 and 5 where they study the interaction with modulating peptides/proteins, run the risk of overinterpreting small differences in band patterns. I would therefore suggest that the data, to the extent possible, is backed up by experiments using alternative techniques. For example, it would be possible to show by gel filtration or pull-downs that the peptides actually interact in the way they are intended to.
5. Finally, with respect to the negative stain EM described in the last paragraph of the results section, it should be possible to collect a small data set of the cross-linked particles shown in Fig. S5b and perform a reference-free 2D classification. Even just 1000 particles could give a good picture of the various views of the particle. This would also allow the authors to lend more support to their conclusion that the complex is indeed conformationally flexible.

Ditlev E. Brodersen
Aarhus University, Denmark

Reviewer #2 (Remarks to the Author):

The manuscript by Raisch et al investigates the following key questions regarding the function of the CNOT deadenylase complex: What are the contributions of each catalytic subunit to CNOT mediated deadenylation? What are the roles and contributions of the non-enzymatic subunits?
This work represents a major technical advance in that the authors have succeeded in reconstituting the human CNOT complex with purified components. They then set about measuring the activity and specificity of the complex using a series of well-executed biochemical assays. As a result, they provide multiple important new insights into the molecular function of CNOT.
The authors report that the full eight subunit CNOT complex is dramatically more active than the two deadenylases, either alone or as a heterodimer. The full CNOT complex also is more specific for poly(A). To interrogate the contributions of individual subunits, the authors reconstitute the CNOT complex with select subunits, while omitting certain others. They observe that CNOT10 and 11 could be omitted without much effect on deadenylation. A minimal 6 subunit complex was also fully active, showing that the CNOT10:11 module and N-terminus of Not1 were not required, nor were disordered regions of CNOT2 and 3. In contrast, deletion of the C-terminal NOT domain and its binding partners CNOT2 and 3 resulted in a substantial decrease in activity. Yet this core complex was still substantially more active than the CCR4:CAF1 heterodimer alone, indicating that CNOT1 and CAF40 promote catalysis. Together, these results provide compelling evidence that multiple CNOT subunits (CNOT2, 3, CAF40, CNOT1) contribute to catalysis, whereas the CNOT10 and 11 subunits did not have much effect in these conditions.
They then investigate the roles of each deadenylase and find that mutation of either does not prevent deadenylation in the context of the heterodimer, though CAF1 activity appears to be less poly(A) specific, since it degraded beyond the poly(A) tail. In the context of the full CNOT complex, CCR4 was poly(A) specific, and mutation of CCR4 greatly reduced the rate of deadenylation. Mutation of CAF1 has a minor effect on rate, but did reduce degradation into non-A sequence. These observations were corroborated with the minimal and core CNOT complexes. These results accentuate the differential contributions of CCR4 and CAF1 to deadenylation activity.
The authors identify multiple CNOT subunits that bind to RNA substrate including CAF40 and NOT10 and 11. They explored the CAF40 RNA interaction and its effect on CNOT activity. They find that CAF40 interacts with RNA versus protein partners in a mutually exclusive manner. The CAF40 binding motif of BAM protein prevents binding to RNA by CAF40 and blocks the ability of CAF40 to promote CNOT mediated deadenylation. This observation supports the contribution of CAF40 to CNOT activity and is interesting because CAF40 is the adapter for sequence specific RNA binding regulators that recruit CNOT to target mRNAs. Interestingly, the authors also show that the CNOT10 and 11 subunits, which they also show bind to substrate RNA, can partly compensate for the inhibitory effect of BAM on CAF40. These observations were corroborated by using two additional CAF40 binding domains from Roquin and CNOT4 RNA binding proteins. Overall these experiments illuminate that contributions of multiple RNA binding modules to CNOT mediated deadenylation.
This report also shows that longer substrate RNA is deadenylated more rapidly by the CNOT complex but not the deadenylase heterodimer. This effect was at least partially dependent on the CNOT10 and 11 subunits, indicating that their RNA binding activity may contribute to recognition of the longer substrate. Single stranded RNA is preferred, as secondary structure diminishes the deadenylation rate.
Finally, the authors perform electron microscopy of the CNOT complex using negative staining and observe that it is conformationally heterogeneous, suggesting that regulators and/or substrates may affect 3D shape of the complex.
Overall this important study is well executed and provides crucial new insights into CNOT function. The biochemical reconstitution and enzymatic analysis are highly significant and timely. The approaches

are state of the art and the resulting data are high quality. The results are concisely presented. Experiments are well controlled. The manuscript is well written. I did not identify typographical errors. Methods and reagents are described in detail.
I do have several suggestions for improvement. First, have the authors investigated the impact of omission of CAF40 on deadenylation?
The authors should consider investigating the affinity and sequence specificity of the RNA-binding activity of CAF40 and CNOT10 and 11. The methods indicate that 25 micromolar protein was used in the EMSA assays, resulting in only partial shift of the ligand RNA. This suggests that the RNA interactions are rather weak compared to regulatory RNA binding proteins that can recruit CNOT to specific target mRNAs, such as Roquin. The authors may wish to comment on this. Still, the impact of these CNOT RNA binding components (CAF40, CNOT10:11) is made clear in the activity assays.
Quantitation and statistics would support the reproducibility of the observations. I understand that the patterns of RNA decay are complex, which makes quantitation more challenging. They should consider measuring the rate of disappearance of substrate, and/or rate of appearance of deadenylated product.
Though the authors do provide a supplementary table that cross references the nonmenclature of subunits, I recommend using the HUGO gene nomenclature CNOT1, 2, 3, 4, 5, 6, 6L, 7, 8, 9, 10, 11.
The authors should indicate the Enzyme:Substrate molar ratio in the figure legends for all panels.

Reviewer #3 (Remarks to the Author):

The authors have achieved a reconstitution of the human CCR4-NOT complex from overproduced and purified subunits. They have performed a first analysis of the contributions of the eight different subunits to the poly(A)-degrading activity of the complex.

Although I find that the authors sometimes tend to overstate their case, the paper is technically very well done, it is quite convincing, and the results are interesting.
I have a number of suggestions for improving the manuscript:

1. Three general comments regarding deadenylation assays:
First, the authors compare different protein preparations. They state (lines 202ff) that all complexes were reconstituted by identical procedures, so functional differences were due to differences in composition, not in procedures. It would be more important if the authors could state that results were reproducible with independent preps of the same type of complex.
Second, the reader is often asked to compare results in different figures. As the differences are frequently not terribly big, one would like to know that the differences were seen in side-by-side comparisons (even though that is not how they are presented).
Third, in their comparisons of different protein complexes or different substrates, the authors use qualitative expressions, like ‚drastically enhanced‵ etc. Can they please provide approximate numbers instead? I know that the assays are hard to quantify, but there would be ways of making estimates. (What the authors consider ‚drastically enhanced‵ in line 328 looks like 2-3fold to me. Should one call that ‚drastic‵?)

2. Fig.2b and c and corresponding text: It appears that the difference is more than fivefold (i. e. more than the difference in protein amounts).
Lines 158ff: CCR4-NOTfull was ‚much more selective for poly(A)‵. The difference between Fig. 2c and d, to which the authors refer, reflects the ability of the proteins to take off a single U residue before they enter another run of three As. The sweeping conclusion drawn by the authors seems a bit overstated.

3. Fig. 2h and corresponding text: The protein concentration used here is very high (25 micromolar; line 662). This should be pointed out in a more prominent location.

With a non-specific assay like this, and in particular at such high protein concentrations, one has to worry about a role of contaminating proteins in the activity. Controls should be performed to exclude this, if possible. For example, does the activity co-migrate with the protein in a column?
In contrast to the deadenylation assays, the exact protein complexes used for this figure are not well described. Put the information into the main text or the figure legend. (E. g. does the NOT10:NOT11 module include the relevant domain of NOT1?)
I assume the conclusion (line 411) that the NOT module does not bind RNA is based on this figure. This is a negative result in a non-equilibrium binding assay, so it should be interpreted with caution.
The claim that the NOT10:NOT11 module ‚binds RNA..... with the highest relative affinity...' (lines 419f) sounds a bit overstated given that a single data point is shown and that the assays has its technical limitations.

4. Fig. S3 and text (lines 237ff): The data support the conclusion that CCR4a is more active than CAF1 (line 239), but they do not permit any conclusion regarding poly(A) specificity, as the complexes with mutant CCR4 do not digest far enough that this question could be judged; longer time courses would be needed.

5. In the experiments examining the Bam CBM, indicate molar concentrations of the molecule, not molar equivalents; presumably, the titrations are equivalent to KD determinations, so concentrations are important, not molar ratios.
Fig. S4c: One cannot judge the inhibition or lack of it since no comparable time-course in the absence of inhibitor is provided. Point out that the concentration of Bam CBM in this experiment was very high.
The term ‚equivalent saturating concentrations of Bam CBMMut' is inappropriate, as the mutant does not inhibit, thus it does not saturate (at least not at the concentrations examined).
From a comparison of Fig. 4b and 4d, the authors conclude (line 287) that CCR4-NOTmini (4d) was inhibited by Bam CBM less efficiently than CCR4-NOTcore (4b). I am not sure. The uninhibited activity of core only progresses to the middle of the poly(A) sequence, whereas mini deadenylates almost to completion; I do not think that the extent of inhibition can be compared under these conditions. Because uninhibited activity goes almost to completion, the comparison of 4d, 4e and 4f is also a bit problematic. However, since Figs. 2 and 5 show that all three protein complexes have very similar activities, I am willing to buy the authors' conclusions.
It is a bit unfortunate that the Bam CBM titrations do not start at 0. However, since the MBP controls show no concentration-dependent inhibition, they can be taken as the missing zero sample.

6. The authors suggest that NOT10 and/or NOT11 also bind RNA. They might wish to do a quick UV X-link experiment.

7. Fig. 6 and corresponding text:
Time points in 6a and c are not well chosen; deadenylation is almost complete at the first time point.
The authors conclude that longer sequences 5' of poly(A) permit more efficient deadenylation (see also Discussion). This conclusion is not terribly safe since they only test two different sequences (6d and e), which are not so different after all (sequences different only in the last seven nt upstream of poly(A); both are AU-rich and lack any G; remaining 13 nt are identical).
Line 334f: ‚These results suggest...' – The logic of this sentence, in particular with respect to the preceding sentence, is not entirely clear.

8. Lines 396ff: Please explain from which data you conclude that a stretch of more than two non-A residues is needed to block the enzyme. As far as I could tell, none of the substrates tested permits this conclusion. The fact that UCU accumulates as an end product with the substrate used most of the time could simply reflect the length limit of digestion.

9. The fact that linear motifs from NOT-binding proteins compete with RNA binding of CAF40 is interesting, but the functional relevance remains a matter of speculation. For my personal taste, the

discussion of these observations might be toned down a bit.

10. I have not looked specifically at splice variants of the subunits of CCR4-NOT, but likely there are quite a few. Please be as precise as possible what exactly was cloned and overproduced (accession numbers or so).

Minor points:

I have a few complaints about the first paragraph of the Introduction, which discusses features of poly(A) tails:
It would make sense to state that the poly(A) tail enhances translation initiation and affects mRNA decay by inhibition of cap hydrolysis.
To say that tails are ‚shortened to 60-80 nt' is a bit misleading; I believe this is the average steady-state length of poly(A) tails, not some limit of shortening.
‚Highly translated mRNAs have relatively short poly(A) tails.' This has indeed been observed, but there is, to my knowledge, no mechanistic explanation and the observation is not very relevant for the paper. Leave it out?
‚CCR4-NOT complex is a master regulator of gene expression.' This term has been used in the literature, but is this not an exaggeration? My feeling is (I may be wrong) that the functions proposed for the complex beyond deadenylation have not been well substantiated.
Line 46: Replace ‚degradation' by ‚deadenylation'.

Lines 119ff: From the description of the NOT11 construct, it appears that the claim of a ‚complete' reconstitution is only about 95% true. Since the function of NOT10/11 is discussed extensively, this limitation should be mentioned, e. g. in the Discussion.

Line 167: This is a matter of taste, but I suggest the authors delete the whole line and simply say ....'if we could delineate....' – line 167 sounds pompous.

Lines 177ff: Break down the sentence into pieces, it is too long.

Line 195: Start the sentence by saying that you are summarizing the results so far.

Lines 202ff: The remark starting by ‚Importantly...' refers to deadenylation assays, but it is made after the description of RNA binding assays. Move it to a better position.

Line 210: ‚In yeast...'. This is a bit misleading, as one tends to think of S. cerevisiae and in vivo assays. However, the experiments described were done in vitro with proteins from S. pombe. (This is important as S. cerevisiae Caf1 is thought to be inactive anyway.)

Line 213: The authors cite ref. 48 and say that ‚the human CCR4a:CAF1 heterodimer.... is inactive....'. However, their own data described in the next paragraph do not confirm the claim in Ref. 48. Thus, it would be appropriate to say something cautious like ‚has been reported to be inactive' or so. The authors should also bluntly state that their own results are different.

Lines 215ff: The results described in this paragraph would be more simply summarized by a statement like ‚CCR4 is more specific for A residues than CAF1'.

Line 317: Insert ‚and' at the end of the line.

Line 374: Insert ‚Our' before ‚studies' to make clear that you are not longer referring to published data, but summarizing your own results.

A point-by-point response to the reviewers' comments

Reviewer #1:

Raisch et al. present a highly interesting and very detailed study of the function of the human CCR4-NOT complex based mainly on in vitro degradation of RNA. Using carefully assembled complexes and sub-complexes, the authors are able to dissect the separate catalytic and RNA-binding functions of the complex and start to answer some of the many questions regarding this important deadenylation complex.

The purification and reconstitution of the human CCR4-NOT complex as presented in this paper constitutes a monumental and very impressive accomplishment for which the authors should be acknowledged. Several of the proteins involved contain unstructured regions and handling these as well as reconstitution of such a large, eukaryotic protein complex is in no way trivial. It provides the authors with a unique tool to study the activity and RNA-binding capabilities of CCR4-NOT in vitro, as well as work with subcomplexes and mutants that have never before been studied.

The manuscript is overall very well written and the figures are generally clear and beautifully laid out. That being said, my main concern with the manuscript is that it relies almost exclusively on a single technique, namely in vitro RNA degradation assays using defined, 5' fluorescently labelled RNA substrates. While this technique can give useful insights into specific preferences for RNA sequence and structure, it also involves a risk of over-interpretation of subtle changes in degradation patterns that may or may not have biological relevance.

Major issues

1. The first thing that strikes me is that the model RNA used only contains a "body" (i.e. non-poly-A part) of 7 nucleotides, which is very short given the relatively large size of the CCR4-NOT complex. I think it is possible that the complex might recognise or interact with non-poly-A sequence outside of this and would like the authors to confirm experimentally that other model RNAs with longer bodies behave in a similar fashion to the one used here. For example, when (on page 8) the authors conclude that the NOT10:NOT11 module but not the NOT module interacts with the 7-mer-A20 RNA, would that be true also for a longer model RNA?

We apologize that this was not clear. As shown in Figure 6 and the new Supplementary Figure 5, we have actually used three (now four) different RNA substrates with longer bodies than the 7-mer. For all longer substrates, we observe an enhancement of deadenylation compared to the short 7-mer-A_{20} substrate. We also show for one of these substrates that this increase in deadenylation efficiency may be attributed to the non-catalytic modules of the complex because the CCR4a:CAF1 dimer did not discriminate between short and long substrates in deadenylation assays (please see Figure 2c vs 6b).

We have also performed two additional EMSA experiments where we tested the binding of the short 7-mer-A_{20} RNA and the long 13+7-mer-A_{20} RNA, respectively, to the individual CCR4-NOT modules as well as the CCR4-NOT_{FULL} complex at lower protein concentrations (1 µM) compared to the previous experiments. Long and short RNA substrates both interact exclusively with the NOT10:NOT11 heterodimer and the CCR4-NOT_{FULL} complex, but not with any of the other subcomplexes under these conditions (please see Figure 2h and Supplementary Figure 5a).

2. When working with RNA and purified nucleases there is always the risk of co-purifying small amounts of endogenous nucleases from the expression host, whether E. coli or insect cells. I would therefore like to see a control in Fig. 2b where both active sites of CCR4 and Caf1 have been inactivated showing that in that case there is no RNA degradation. This can easily be achieved by combination of the protein samples shown in Fig. 3.

We thank the reviewer for raising this concern (please also see also comment by reviewer #3). To address the point about potential purification contaminants contributing to the observed deadenylation activity, we have performed deadenylation assays with a CCR4a:CAF1 dimer in which both enzymatic activities are inactivated by mutations in the active sites. This mutant nuclease dimer demonstrates no detectable deadenylation activity either in isolation or when incorporated into the CCR4-NOT_{FULL} and CCR4-NOT_{MINI} reconstituted complexes (please see Supplementary Figures 3f-h).

3. Also in Fig. 2, the authors conclude that the activity of the isolated CCR4:Caf1 heterodimer is not very high and that a 5:1 ratio of protein to RNA needs to be used. This might seem extreme for an enzyme to have to be in excess of its substrate, but is indeed what has been observed in vitro for other deadenylases. However, when looking at the mutants in Fig. 3a/b it needs to be clearer what ratio of protein:RNA is used (I couldn't find this in the legend). I assume this is also 5:1, otherwise the experiments are not consistent with Fig. 2.

We apologize for this omission in the labeling of the figures and we thank the reviewer for pointing this out. Indeed, we have used a [5:1] molar ratio in the assays shown in Figures 3a and 3b. We have now provided the information on enzyme/substrate molar ratios and absolute concentrations in the legends for all figures in which the deadenylation assays are shown (please see also comment by reviewer #3).

4. As mentioned above, relying too much on a single technique can be misleading and I think that the authors in the later figures, especially Fig. 4 and 5 where they study the interaction with modulating peptides/proteins, run the risk of overinterpreting small differences in band patterns. I would therefore suggest that the data, to the extent possible, is backed up by experiments using alternative techniques. For example, it would be possible to show by gel filtration or pull-downs that the peptides actually interact in the way they are intended to.

The interaction of all three used peptides with the CCR4-NOT complex has already been extensively characterized in previous studies (Sgromo et al., 2017; Sgromo et al., 2018; Keskeny et al., 2019). These interactions were experimentally validated in pulldowns, co-crystal structures, and functional assays. In the present study, we used this substantial body of knowledge to guide our experimental design. For example, we used structure-guided single-site mutants of the Bam CBM to relieve the inhibition of deadenylation by this peptide and this was confirmed in a deadenylation assay (Figure 4c).

Nonetheless, we agree that it is possible that the interactions in the context of the complete CCR4-NOT perhaps may differ from those observed with subcomplexes in previous studies. To assess this, we carried out a new set of pulldown assays and we observe that the Bam CBM and the Roquin C-terminal fragment interact *in vitro* with the CCR4-NOT_{FULL} and CCR4-NOT_{MINI} complexes. These pulldown assays are now shown in the new Supplementary Figure 4b and 4c.

5. Finally, with respect to the negative stain EM described in the last paragraph of the results section, it should be possible to collect a small data set of the cross-linked particles shown in Fig. S5b and perform a reference-free 2D classification. Even just 1000 particles could give a good picture of the various views of the particle. This would also allow the authors to lend more support to their conclusion that the complex is indeed conformationally flexible.

We have followed the reviewer's suggestion and attempted to perform a reference-free 2D classification by equal-size k-means clustering using SPHIRE on several independent negative stain datasets. However, we were not able to obtain convincing 2D class averages with which we felt we could make some conclusions about shape or conformation. More comprehensive EM analysis of the human CCR4-NOT is indeed an important future goal but one we feel is outside the scope of the present study. The CCR4-NOT is unlikely to be a rigid complex based on what we already know from high-resolution structures of subcomplexes and bioinformatic analyses, which indicates the presence of extensive low-complexity regions linking the structured modules. This, together with the observation that dimensions of particles are similar led us to conclude that the CCR4-NOT is unlikely to adopt a set of discrete conformations but rather exists in a continuum of many different states, which unfortunately precludes reliable 2D classification.

Reviewer #2:

The manuscript by Raisch et al investigates the following key questions regarding the function of the CNOT deadenylase complex: What are the contributions of each catalytic subunit to CNOT mediated deadenylation? What are the roles and contributions of the non-enzymatic subunits?

This work represents a major technical advance in that the authors have succeeded in reconstituting the human CNOT complex with purified components. They then set about measuring the activity and specificity of the complex using a series of well-executed biochemical assays. As a result, they provide multiple important new insights into the molecular function of CNOT.

The authors report that the full eight subunit CNOT complex is dramatically more active than the two deadenylases, either alone or as a heterodimer. The full CNOT complex also is more specific for poly(A). To interrogate the contributions of individual subunits, the authors reconstitute the CNOT complex with select subunits, while omitting certain others. They observe that CNOT10 and 11 could be omitted without much effect on deadenylation. A minimal 6 subunit complex was also fully active, showing that the CNOT10:11 module and N-terminus of Not1 were not required, nor were disordered regions of CNOT2 and 3. In contrast, deletion of the C-terminal NOT domain and its binding partners CNOT2 and 3 resulted in a substantial decrease in activity. Yet this core complex was still substantially more active than the CCR4:CAF1 heterodimer alone, indicating that CNOT1 and CAF40 promote catalysis. Together, these results provide compelling evidence that multiple CNOT subunits (CNOT2, 3,

CAF40, CNOT1) contribute to catalysis, whereas the CNOT10 and 11 subunits did not have much effect in these conditions.

They then investigate the roles of each deadenylase and find that mutation of either does not prevent deadenylation in the context of the heterodimer, though CAF1 activity appears to be less poly(A) specific, since it degraded beyond the poly(A) tail. In the context of the full CNOT complex, CCR4 was poly(A) specific, and mutation of CCR4 greatly reduced the rate of deadenylation. Mutation of CAF1 has a minor effect on rate, but did reduce degradation into non-A sequence. These observations were corroborated with the minimal and core CNOT complexes. These results accentuate the differential contributions of CCR4 and CAF1 to deadenylation activity.

The authors identify multiple CNOT subunits that bind to RNA substrate including CAF40 and NOT10 and 11. They explored the CAF40 RNA interaction and its effect on CNOT activity. They find that CAF40 interacts with RNA versus protein partners in a mutually exclusive manner. The CAF40 binding motif of BAM protein prevents binding to RNA by CAF40 and blocks the ability of CAF40 to promote CNOT mediated deadenylation. This observation supports the contribution of CAF40 to CNOT activity and is interesting because CAF40 is the adapter for sequence specific RNA binding regulators that recruit CNOT to target mRNAs. Interestingly, the authors also show that the CNOT10 and 11 subunits, which they also show bind to substrate RNA, can partly compensate for the inhibitory effect of BAM on CAF40. These observations were corroborated by using two additional CAF40 binding domains from Roquin and CNOT4 RNA binding proteins. Overall these experiments illuminate that contributions of multiple

RNA binding modules to CNOT mediated deadenylation.

This report also shows that longer substrate RNA is deadenylated more rapidly by the CNOT complex but not the deadenylase heterodimer. This effect was at least partially dependent on the CNOT10 and 11 subunits, indicating that their RNA binding activity may contribute to recognition of the longer substrate. Single stranded RNA is preferred, as secondary structure diminishes the deadenylation rate.

Finally, the authors perform electron microscopy of the CNOT complex using negative staining and observe that it is conformationally heterogeneous, suggesting that regulators and/or substrates may affect 3D shape of the complex.

Overall this important study is well executed and provides crucial new insights into CNOT function. The biochemical reconstitution and enzymatic analysis are highly significant and timely. The approaches are state of the art and the resulting data are high quality. The results are concisely presented. Experiments are well controlled. The manuscript is well written. I did not identify typographical errors. Methods and reagents are described in detail.

I do have several suggestions for improvement. First, have the authors investigated the impact of omission of CAF40 on deadenylation?

We have not done this because it is technically impossible in our hands. We fully agree with the reviewer that it would be very interesting and informative to omit the CAF40 subunit from the reconstitution of the complex to study the effects of this on activity. CAF40 is absolutely essential for the correct folding of its cognate binding domain of NOT1 (CN9BD). In the absence of CAF40, this NOT1 CN9BD cannot be stably produced, and this has already been described in a previous study (Chen et al., 2014). Even if it were possible to stabilize somehow in solution it would not be possible to distinguish between the effects of misfolding of NOT1, which acts as the central scaffold of the CCR4-NOT, and the omission of CAF40 on the deadenylation activity. We also note that this would apply even if the CN9BD were completely deleted in the NOT1 construct. This domain of NOT1 is located in the middle of the NOT1 protein and very likely serves to support the structural integrity of the complex.

The authors should consider investigating the affinity and sequence specificity of the RNA-binding activity of CAF40 and CNOT10 and 11. The methods indicate that 25 micromolar protein was used in the EMSA assays, resulting in only partial shift of the ligand RNA. This suggests that the RNA interactions are rather weak compared to regulatory RNA binding proteins that can recruit CNOT to specific target mRNAs, such as Roquin. The authors may wish to comment on this. Still, the impact of these CNOT RNA binding components (CAF40, CNOT10:11) is made clear in the activity assays.

We thank the reviewer for these suggestions. We have now included EMSA assays with the individual modules and the CCR4-NOT$_{FULL}$ complex at 1 µM concentration. Under these conditions, we observe RNA binding with the CCR4-NOT$_{FULL}$ and the NOT10:NOT11 heterodimer. This was tested with the 7-mer-A$_{20}$ (new Figure 2h) and the 13+7-mer-A$_{20}$ (new Supplementary Figure 5a) RNA substrates. Indeed, the CAF40 module no longer interacts stably with either of these RNA substrates at this lower concentration in contrast to detectable binding at 25 µM (new Supplementary Figures 2e and 5b).

We have tested the binding of CCR4-NOT$_{FULL}$ to four different homopolymeric 30-mer RNAs in EMSA to examine possible sequence preference. The CCR4-NOT binds poly(U) and poly(G) sequences, whereas binding to poly(A) and poly(C) is significantly weaker (new Supplementary Figure 2g). This does suggest that the CCR4-NOT can exhibit some sequence preferences via its RNA-binding modules.

Furthermore, we have attempted to measure binding affinities of all modules as well as the CCR4-NOT$_{FULL}$ and CCR4-NOT$_{MINI}$ complexes for the 7-mer-A$_{20}$ and 13+7-mer-A$_{20}$ RNAs under equilibrium conditions by using fluorescence polarization and microscale thermophoresis (please see also the response to reviewer #3). However, we were not able to reach the very high protein concentrations necessary to saturate the RNA substrate with the isolated modules in solution in order to perform reliable curve fitting to derive binding constants.

Quantitation and statistics would support the reproducibility of the observations. I understand that the patterns of RNA decay are complex, which makes quantitation more challenging. They should consider measuring the rate of disappearance of substrate, and/or rate of appearance of deadenylated product.

We have very carefully considered this point (please see also the response to reviewer #3). We agree that it is more rigorous to use quantitation of product formation or substrate disappearance when assessing enzymes *in vitro*. This would be quite straightforward in a situation where there was a direct conversion of substrate to product. Here, it is rather more complex. In these deadenylation assays, it is rather more informative to assess the full complexity of the mechanism via direct visualization of the primary data. Representing deadenylation assays with a number such as nt/sec would be technically feasible via estimates based on quantization of band intensities but this may not give an accurate representation as, for example when two different deadenylation products accumulate. It would be rather confusing to the readers who may not be familiar with all the nuances of deadenylation kinetics to be presented with two or three estimated metrics in the case of some complexes and not with others. We are rather of the opinion that the gels are intuitively more understandable to the wider readership.

As to the reproducibility, obtaining a consistent and reproducible *in vitro* deadenylation system is the *raison d'être* of this study. Our motivation in reconstituting the human CCR4-NOT from recombinant and purified components was to obtain material that would reproducibly exhibit the same activity across different batches purified at different times in a strictly defined *in vitro* scenario. All assays are supported by technical as well as biological replicates, which means that we had verified the result

with complexes reconstituted at different times and with different batches of protein. The assays are exquisitely reproducible up to the individual time points in our hands and were validated extensively.

We also point to previous studies by the Passmore group and others where the *in vitro* deadenylation analysis of the Ccr4-Not from fission yeast was presented rather as we have here – in a form of synthetic deadenylation products separated on TBE-urea gels.

Though the authors do provide a supplementary table that cross references the nomenclature of subunits, I recommend using the HUGO gene nomenclature CNOT1, 2, 3, 4, 5, 6, 6L, 7, 8, 9, 10, 11.

We thank the reviewer for pointing out this issue. Regrettably, this topic is generally problematic within the RNA decay field. On the one hand, the human proteins are, as the reviewer points out, named according to HUGO as CNOTx. However, we feel this is confusing as there is a clear discrepancy to the nomenclature in yeast where the proteins are named Not1-5, Caf1, Caf40, and Ccr4. In this study, we draw comparisons across species and to make these more accessible to the wider readership we have endeavored to simplify and to reduce the nomenclature overload in the text. We also note that alternative gene/protein names for the CCR4-NOT components are used interchangeably in HUGO as well as in UniProt databases. To prevent any confusion, we have now added the HUGO names of all subunits in the "Synonyms" column in Supplementary Table 1.

The authors should indicate the Enzyme:Substrate molar ratio in the figure legends for all panels.

We apologize for this omission in the labeling of the figures and we thank the reviewer for pointing this out. All the necessary information is now provided (please also see the response to reviewer #1).

Reviewer #3

The authors have achieved a reconstitution of the human CCR4-NOT complex from overproduced and purified subunits. They have performed a first analysis of the contributions of the eight different subunits to the poly(A)-degrading activity of the complex.

Although I find that the authors sometimes tend to overstate their case, the paper is technically very well done, it is quite convincing, and the results are interesting.

I have a number of suggestions for improving the manuscript:

1. Three general comments regarding deadenylation assays:

First, the authors compare different protein preparations. They state (lines 202ff) that all complexes were reconstituted by identical procedures, so functional differences were due to differences in composition, not in procedures. It would be more important if the authors could state that results were reproducible with independent preps of the same type of complex.

We also consider this issue to be of overriding importance to the integrity and reproducibility of this study. We tested not only different preparations obtained independently and at different times, but also with different baculovirus and at the hands of several authors of the study. We found the deadenylation assays to be remarkably consistent and reproducible to the level of individual time points in assays with different, independently purified and reconstituted batches of complexes. We have added a statement explicitly to this effect in the Results and Methods sections of the manuscript as suggested by the reviewer (please see also the response to reviewer #2).

Second, the reader is often asked to compare results in different figures. As the differences are frequently not terribly big, one would like to know that the differences were seen in side-by-side comparisons (even though that is not how they are presented).

We thank the reviewer for raising this concern and we agree that it would make comparison much easier if assays were presented side by side. Unfortunately, since it is not possible to fit all assays that would need to be compared in one single figure, we would need to duplicate some panels and show them in multiple figures in order to make such an arrangement. Even though this might be possible in

principle, we are rather of the opinion that it may be misleading to show the same panel multiple times. We would, therefore, prefer to keep the order and arrangement as it is now. But we have indeed seen all the assays side by side in many multiple configurations before settling on the current figure arrangement.

Third, in their comparisons of different protein complexes or different substrates, the authors use qualitative expressions, like ‚drastically enhanced' etc. Can they please provide approximate numbers instead? I know that the assays are hard to quantify, but there would be ways of making estimates. (What the authors consider ‚drastically enhanced' in line 328 looks like 2-3fold to me. Should one call that ‚drastic'?)

Please also see our response to reviewer #2 regarding quantitation. We feel that single metric representations may indeed appear more satisfying and give an initial impression of rigor but would mask the complexity of the deadenylation patterns where the appearance of several products at different time points is rather the norm than the exception. In this, we were also guided by the convention established in previous studies of deadenylation by recombinant Ccr4-Not complexes (see, for example, Stowell et al., 2016 and Webster et al., 2018) where deadenylation by different complexes was presented rather as we have it here.

We removed the term “drastically”.

2. Fig.2b and c and corresponding text: It appears that the difference is more than fivefold (i. e. more than the difference in protein amounts).

We fully agree with the reviewer that the concomitant increase in the rate appears to be greater than the increase in the molar ratio, but since it is technically difficult to reliably quantify the rates of these multistep reactions (please see also our response to the previous question), we do not want to make any strong claims based on this. However, we have diligently followed up on this and tested the effect of increasing the protein-RNA ratio from 1:1 to 5:1 on the deadenylation rate (please see the Supplementary Figure 2c).

Lines 158ff: CCR4-NOTfull was ‚much more selective for poly(A)'. The difference between Fig. 2c and d, to which the authors refer, reflects the ability of the proteins to take off a single U residue before they enter another run of three As. The sweeping conclusion drawn by the authors seems a bit overstated.

We thank the reviewer for raising this concern. The observed difference in the deadenylation patterns in Figures 2c and 2d does appear to be rather significant as the CCR4-NOT$_{FULL}$ complex in 2d does seem to stall on encountering the first non-A residue in comparison to the isolated exonucleases in 2c. This apparent selectivity for polyadenosine for the CCR4-NOT$_{FULL}$ complex has also been verified with different substrates as shown in Figure 6. However, we take the point and we have replaced “much more selective” by “appeared to be more selective” in the text.

3. Fig. 2h and corresponding text: The protein concentration used here is very high (25 micromolar; line 662). This should be pointed out in a more prominent location.

Please also see our response to reviewer #2 regarding concentrations and affinities. We agree with the reviewer that the concentration is very high and have now indicated the used concentrations also in the legend of Supplementary Figure 2e (which has replaced the previous Figure 2h) and the new Supplementary Figure 5b. In addition, we have performed EMSA experiments at a lower protein concentration of 1 µM and we observe that under these conditions only the NOT10:NOT11 heterodimer and the CCR4-NOT$_{FULL}$ complex, but not any of the other modules, retain RNA-binding. These new assays are shown in Figure 2h and Supplementary Figure 5a.

With a non-specific assay like this, and in particular at such high protein concentrations, one has to worry about a role of contaminating proteins in the activity. Controls should be performed to exclude this, if possible. For example, does the activity co-migrate with the protein in a column?

In contrast to the deadenylation assays, the exact protein complexes used for this figure are not well described. Put the information into the main text or the figure legend. (E. g. does the NOT10:NOT11 module include the relevant domain of NOT1?)

Please also see our response to reviewer #1 regarding potential purification contaminants contributing to the observed deadenylation activity. We have performed deadenylation assays with a CCR4a:CAF1 dimer in which both enzymatic activities are inactivated by mutations in the active sites. This mutant nuclease dimer demonstrates no detectable deadenylation activity either in isolation or when incorporated into the CCR4-NOT_{FULL} and CCR4-NOT_{MINI} reconstituted complexes (please see Supplementary Figures 3f-h).

We had explicitly described in the figure legend the composition of the complexes tested and we apologize that this was not clear. The precise construct boundaries of complexes investigated are also described in detail in the Supplementary Table 3.

I assume the conclusion (line 411) that the NOT module does not bind RNA is based on this figure. This is a negative result in a non-equilibrium binding assay, so it should be interpreted with caution.

The claim that the NOT10:NOT11 module ‚binds RNA..... with the highest relative affinity...' (lines 419f) sounds a bit overstated given that a single data point is shown and that the assays has its technical limitations.

We thank the reviewer for pointing out the limitations of the EMSA (please also see our response to reviewer #2 regarding our attempts to characterize binding under equilibrium conditions). We have now added a *caveat emptor* in the text that pays heed to the reviewer's excellent point that the absence of evidence of RNA binding by the NOT module is not evidence of absence.

Regarding the relatively high affinity of the NOT10:NOT11 module, we have now performed new EMSA experiments at lower protein concentrations (1 µM compared to the previous 25 µM), and under these conditions we only see the NOT10:NOT11 heterodimer, but not the CAF40 module or any of the other modules, binding to both the 7-mer-A_{20} and the 13+7-mer-A_{20} RNA substrates (please see new Figure 2h and Supplementary Figure 5a). From this we tentatively conclude that under these non-equilibrium conditions the NOT10:NOT11 module does appear to display the highest relative RNA-binding affinity of all the CCR4-NOT modules we tested under the same conditions.

4. Fig. S3 and text (lines 237ff): The data support the conclusion that CCR4a is more active than CAF1 (line 239), but they do not permit any conclusion regarding poly(A) specificity, as the complexes with mutant CCR4 do not digest far enough that this question could be judged; longer time courses would be needed.

We thank the reviewer for raising this concern. To address this, we have performed deadenylation assays with the catalytic mutants of either CAF1 or CCR4a in the context of the CCR4-NOT_{MINI} complex for up to 3 hours. We have included these results in the new Supplementary Figure 3c. Strikingly, the complex carrying wildtype CCR4a and the catalytically inactive CAF1 shows a high degree of specificity for polyadenosine as it stops at A_1, while the complex with the CCR4a mutant, despite some preference for polyadenosine, is still able to degrade the substrate completely to the terminal UCU trinucleotide.

5. In the experiments examining the Bam CBM, indicate molar concentrations of the molecule, not molar equivalents; presumably, the titrations are equivalent to KD determinations, so concentrations are important, not molar ratios.

We had opted to indicate the molar equivalents instead of absolute concentrations to aid readability. However, on reflection, we agree with the reviewer that this comes at the cost of losing some accuracy. In the revised version, we have therefore indicated absolute concentrations and we thank the reviewer for pointing this out.

Fig. S4c: One cannot judge the inhibition or lack of it since no comparable time-course in the absence of inhibitor is provided. Point out that the concentration of Bam CBM in this experiment was very high.

The term ‚equivalent saturating concentrations of Bam CBMMut' is inappropriate, as the mutant does not inhibit, thus it does not saturate (at least not at the concentrations examined).

We apologize that this was not clear. The control time course in the absence of the Bam peptide is shown in Figure 2. We have also added this information to the legend of the Supplementary Figure 4e.

The Bam CBM concentrations were chosen such that the peptide was at 50-fold molar excess over the exonuclease dimer or the CCR4-NOT complex. We have added a statement explaining the reason for the use of high concentration of the Bam CBM in the legend of the Supplementary Figure 4.

We have replaced the term ‘equivalent saturating concentrations’ by a more appropriate phrase in the main text. We thank the reviewer for pointing out the flaw in the logic of the original statement.

From a comparison of Fig. 4b and 4d, the authors conclude (line 287) that CCR4-NOTmini (4d) was inhibited by Bam CBM less efficiently than CCR4-NOTcore (4b). I am not sure. The uninhibited activity of core only progresses to the middle of the poly(A) sequence, whereas mini deadenylates almost to completion; I do not think that the extent of inhibition can be compared under these conditions. Because uninhibited activity goes almost to completion, the comparison of 4d, 4e and 4f is also a bit problematic. However, since Figs. 2 and 5 show that all three protein complexes have very similar activities, I am willing to buy the authors‘ conclusions.

We agree with the reviewer that it is not easy to compare the extent of inhibition the Bam CBM has on the different complexes under exact time course conditions.

It is a bit unfortunate that the Bam CBM titrations do not start at 0. However, since the MBP controls show no concentration-dependent inhibition, they can be taken as the missing zero sample.

We agree with the reviewer that including a control sample without MBP or MBP-CBM in these assays is most optimal but, as the reviewer points out, the MBP controls substitute well under these conditions.

6. The authors suggest that NOT10 and/or NOT11 also bind RNA. They might wish to do a quick UV X-link experiment.

We thank the reviewer for this excellent suggestion. We have now performed UV crosslinking experiments to verify that the NOT10:NOT11 heterodimer and the CCR4-NOT$_{FULL}$ directly bind to the poly(U) RNA. We also observe weaker RNA binding by CAF40 and the exonuclease module; the results of these experiments are now included in the Supplementary Figure 2f.

7. Fig. 6 and corresponding text:

Time points in 6a and c are not well chosen; deadenylation is almost complete at the first time point.

The authors conclude that longer sequences 5‘ of poly(A) permit more efficient deadenylation (see also Discussion). This conclusion is not terribly safe since they only test two different sequences (6d and e), which are not so different after all (sequences different only in the last seven nt upstream of poly(A); both are AU-rich and lack any G; remaining 13 nt are identical).

We thank the reviewer for raising this concern and we agree that the chosen time points in Figure 6a and 6c alone are not sufficient to conclude that the CCR4-NOT deadenylates longer substrates more efficiently. Initially, we chose these time points to draw an effective comparison with the assays performed with the shorter substrates where these time points were also used.

To increase granularity and to better observe the intermediates we did the same assay as in Figure 2 but with the finer sampling of time points (compare Figure 6a with 6d). We apologize that this may not have been clear and we have modified the text to better draw the attention of the reader to this.

We have now performed additional experiments with another RNA containing several Gs as suggested by the reviewer. The result is shown in the new Supplementary Figure 5d and it does seem that the CCR4-NOT$_{FULL}$ complex also pauses when it encounters the first G analogous to the situation when reaching the first C of the substrate in Figure 6e.

Line 334f: ‚These results suggest...‘ – The logic of this sentence, in particular with respect to the preceding sentence, is not entirely clear.

We thank the reviewer for pointing this and we have modified the text accordingly.

8. Lines 396ff: Please explain from which data you conclude that a stretch of more than two non-A residues is needed to block the enzyme. As far as I could tell, none of the substrates tested permits this conclusion. The fact that UCU accumulates as an end product with the substrate used most of the time could simply reflect the length limit of digestion.

We apologize for not making it clear that the final degradation products of the longer RNA substrates in Figure 6 are not a UCU trinucleotide. The product of deadenylation with the 13-US-7-mer-A20 substrate is a species that consists of the UCU trinucleotide **plus** the additional 13 nucleotides at the 5’ end (Figure 6a-d). This is consistent with the product of deadenylation of the other linear substrate, the 20-mer-A20 RNA, where the complex first stalls at the first two consecutive non-A nucleotides and then stalls again when it reaches the next non-A nucleotide.

To make the identities of the products clear, we have now placed labels next to the lowest bands in the marker lanes in all assays shown in Figure 6 and Supplementary Figure 5. In addition, we have added an explanatory sentence in the Results.

9. The fact that linear motifs from NOT-binding proteins compete with RNA binding of CAF40 is interesting, but the functional relevance remains a matter of speculation. For my personal taste, the discussion of these observations might be toned down a bit.

As advised by the reviewer, we have toned down that part of the Discussion and endeavored to make the speculative statements more clearly denoted as such.

10. I have not looked specifically at splice variants of the subunits of CCR4-NOT, but likely there are quite a few. Please be as precise as possible what exactly was cloned and overproduced (accession numbers or so).

We thank the reviewer for pointing this out. We used the respective isoform 1 as annotated in UniProt for the production of the CCR4-NOT subunits. We have added this information in the accession numbers in the Supplementary Table 1.

Minor points:

I have a few complaints about the first paragraph of the Introduction, which discusses features of poly(A) tails: It would make sense to state that the poly(A) tail enhances translation initiation and affects mRNA decay by inhibition of cap hydrolysis.

To say that tails are ‚shortened to 60-80 nt‘ is a bit misleading; I believe this is the average steady-state length of poly(A) tails, not some limit of shortening.

‚Highly translated mRNAs have relatively short poly(A) tails.‘ This has indeed been observed, but there is, to my knowledge, no mechanistic explanation and the observation is not very relevant for the paper. Leave it out? ‚CCR4-NOT complex is a master regulator of gene expression.‘ This term has been used in the literature, but is this not an exaggeration? My feeling is (I may be wrong) that the functions proposed for the complex beyond deadenylation have not been well substantiated.

We thank the reviewer for pointing this and we have modified the text accordingly.

Line 46: Replace ‚degradation‘ by ‚deadenylation‘.

Done.

Lines 119ff: From the description of the NOT11 construct, it appears that the claim of a ‚complete‘ reconstitution is only about 95% true. Since the function of NOT10/11 is discussed extensively, this limitation should be mentioned, e. g. in the Discussion.

We have included a statement that the NOT11 construct is missing the unstructured N-terminal half in the first paragraph of the Discussion as suggested by the reviewer.

Line 167: This is a matter of taste, but I suggest the authors delete the whole line and simply say ....‘if we could delineate....‘ – line 167 sounds pompous.

Done.

Lines 177ff: Break down the sentence into pieces, it is too long.

Done.

Line 195: Start the sentence by saying that you are summarizing the results so far.

We thank the reviewer for pointing this and we have modified the text accordingly.

Lines 202ff: The remark starting by ‚Importantly...‘ refers to deadenylation assays, but it is made after the description of RNA binding assays. Move it to a better position.

We thank the reviewer for pointing this out. We have now moved these two sentences to precede the description of the RNA binding assays.

Line 210: ‚In yeast...‘. This is a bit misleading, as one tends to think of S. cerevisiae and in vivo assays. However, the experiments described were done in vitro with proteins from S. pombe. (This is important as S. cerevisiae Caf1 is thought to be inactive anyway.)

We thank the reviewer for pointing this and we have modified the text accordingly.

Line 213: The authors cite ref. 48 and say that ‚the human CCR4a:CAF1 heterodimer.... is inactive....‘. However, their own data described in the next paragraph do not confirm the claim in Ref. 48. Thus, it would be appropriate to say something cautious like ‚has been reported to be inactive‘ or so. The authors should also bluntly state that their own results are different.

We thank the reviewer for pointing this and we have modified the text accordingly.

Lines 215ff: The results described in this paragraph would be more simply summarized by a statement like ‚CCR4 is more specific for A residues than CAF1‘.

We thank the reviewer for pointing this and we have modified the text accordingly.

Line 317: Insert ‚and‘ at the end of the line.

Done.

Line 374: Insert ‚Our‘ before ‚studies‘ to make clear that you are not longer referring to published data, but summarizing your own results.

Done.

REVIEWERS' COMMENTS:

Reviewer #1 (Remarks to the Author):

The authors have answered my queries in a satisfactory way.

Reviewer #2 (Remarks to the Author):

The revised manuscript adequately addressed my original critiques.

Reviewer #3 (Remarks to the Author):

This was a very good manuscript to begin with, and I am fully satisfied with the authors' response to my criticism.
I recommend publication.
